# Ferroelectret nanogenerators for the development of bioengineering systems

**DOI:** 10.1016/j.xcrp.2023.101388

**Published:** 2023-04-24

**Authors:** Wei Li, Yunqi Cao, Chuan Wang, Nelson Sepúlveda

**Affiliations:** 1 Department of Mechanical Engineering, University of Vermont, Burlington, VT 05405, USA; 2 College of Integrated Circuit Science and Engineering, Nanjing University of Posts and Telecommunications, Nanjing, Jiangsu 210023, China; 3 State Key Laboratory of Industrial Control Technology, College of Control Science and Engineering, Zhejiang University, Hangzhou, Zhejiang 310027, China; 4 Electrical and Systems Engineering, Washington University in St. Louis, St. Louis, MO 63130, USA; 5 Institute of Materials Science and Engineering, Washington University in St. Louis, St. Louis, MO 63130, USA; 6 Department of Electrical and Computer Engineering, Michigan State University, East Lansing, MI 48824, USA

## Abstract

Bioengineering devices and systems will become a practical and versatile technology in society when sustainability issues, primarily pertaining to their efficiency, sustainability, and human-machine interaction, are fully addressed. It has become evident that technological paths should not rely on a single operation mechanism but instead on holistic methodologies that integrate different phenomena and approaches with complementary advantages. As an intriguing invention, the ferroelectret nanogenerator (FENG) has emerged with promising potential in various fields of bioengineering. Utilizing the changes in the engineered macro-scale electric dipoles to create displacement current (and vice versa), FENGs have been demonstrated to be a compelling strategy for bidirectional conversion of energy between the electrical and mechanical domains. Here we provide a comprehensive overview of the latest advancements in integrating FENGs in bioengineering systems, focusing on the applications with the most potential and the underlying current constraints.

## INTRODUCTION

As a multidisciplinary research field that integrates the principles of biology and engineering, bioengineering is constantly advanced by the most recent discoveries and novel ideas in electrical and mechanical engineering, material science, chemistry, biology, and other fields of science and technology. The main goal of advancements in bioengineering is to enhance the standard of living for humanity through a holistic approach that yields potent instruments for preserving wellness and combating illness. Bioengineering has become an important driving force behind the rapid advancement of disease prevention and patient treatment because of the ability of electrical and mechanical devices to precisely detect physiological signals coming from patient bodies and process them appropriately.^1^ In the trend of the Internet of Everything (IoE), which expands the emphasis on machine-to-machine communication in the Internet of Things (IoT) to include people and processes as well, the concept of personalized healthcare is rapidly gaining ground to improve therapeutic efficacy, conserve medical resources, and reduce costs.^2,3^ For healthy individuals, wearable bioelectrical devices can be used to identify chemical or physical reactions brought by biological behavior and convert the phenomena into electrical signals.^4–8^ By continuously monitoring, collecting, and analyzing signals from the human body, personalized healthcare anticipates and possibly prevents disease onset.^9^ Rapid progress in artificial intelligence (AI), Big Data, and 5G/6G provides a golden opportunity for fast development of lightweight, miniaturized, flexible, and multifunctional wearable devices that enable comfortable, safe, and novel communication between humans and external devices. The level of convenience in people’s daily lives could increase because of these developments.

Over the past decade, the generation of electricity by exploiting different types of human body-related energies, particularly pervasive and continuous biomechanical energy, has attracted much enthusiasm from researchers. By this effort, numerous devices have been developed that target various application scenarios and have a range of functionalities. A popular and extensively investigated route for biomechanical energy conversion and biosensing is the use of triboelectric nanogenerators (TENGs), which take advantage of triboelectrification, a commonly occurring phenomenon that has been known for about 2,600 years.^10^ The concept can be described as the process of moving static charge that has been generated as the result of the contact between surfaces of dissimilar materials.^11^ In addition, piezoelectric effects, which were discovered about 150 years ago, have been used for several decades in energy harvesting and biosensing.^12,13^ Wang and Song^14^ defined the name “piezoelectric nanogenerators (PENGs)” after they discovered the piezoelectricity in zinc oxide nanowires in 2006. In general, the studies on PENGs focus on using nanostructured piezoelectric materials (e.g., zinc oxide [ZnO], lead zirconate titanate [PZT], and barium titanate) that have molecular dipoles for electricity generation.^15–17^ Ferroelectret nanogenerators (FENGs) mark an important advancement in the piezoelectricity-based conversion of energy. The origin of FENGs can be traced back to a patent filed in 1984.^18^ In contrast to PENGs, FENGs are completely nonpolar unless their internal voids are charged by dielectric barrier microdischarges.^19–21^ Internal artificial voids are usually the hallmark of FENGs, and plasma discharge of these voids makes non-polarized materials behave like piezoelectric materials, where molecular dipoles are intrinsic to the material.^22^ FENGs behave similarly to traditional, well-known piezoelectric polymers or piezocomposite at the macroscopic level, but their microstructural formation and operation principles are different. Ferroelectret materials are also distinguished from conventional piezoelectric materials by their high piezoelectric coefficient and low Young’s modulus, which are derived from the charged cellular structures that make them more sensitive to stress and effective at storing charge. Because of their special physical principles, FENGs combine the merits of the high piezoelectricity observed in inorganic piezoelectric compounds (e.g., PZT) and the flexible thin-film configuration featured by organic piezoelectric polymers (e.g., polyvinylidene fluoride [PVDF]). It is worth noting that, although the appearance of FENGs and TENGs in the vertical contact-separation mode of operation^9^ might be similar under some circumstances, they possess distinct differences. FENGs mimic the properties of piezoelectric materials, while TENGs operate through the mechanism of triboelectrification. In terms of performance, FENGs demonstrate positive and negative piezoelectric effects with comparable efficiency.^23^ The structural composition of FENGs is characterized by foam-like configurations with numerous microscopic voids, whereas TENGs usually feature two electrodes separated by a relatively larger gap. Despite the fact that some FENGs developed recently are self-polarized, most FENGs require a high-voltage polarization process, in contrast to TENGs, which do not require polarization.

To date, the FENG has been proven to be an effective, affordable, lightweight, dependable, and compatible solution for mechanical energy harvesting and self-powered sensing at a broad range of frequencies.^23–30^ As an emerging technology for electromechanical energy conversion, FENGs have been used to produce electricity from diverse biomechanical motions such as arm movement, breathing, vocal vibration, walking, heart beating, pulse wave, blood flow, pressure from blood vessels, stomach peristalsis, etc.^31–33^ Not only can FENGs serve as energy transformers, but they can also be used as bioinformation carriers, bridging the communication gap between people and devices, systems, and machines. Especially with the help of the quick development of IoE and AI, advances in human-machine interface (HMI) offer FENGs an advantageous application scenario in bioengineering. Demonstrations of implementing FENGs in streaming bioinformation between machine and ser include keyboards, microphones, loudspeakers, fitness trackers, etc.^23,34–36^ Additionally, the expansion of implantable and wearable electronics as well as biosensors in the context of IoE is indispensable for personalized healthcare and HMI. Battery-free (self-power) and flexibility are added features that increase the practicality of the implementation of various devices in bioengineering applications. To this end, FENGs’ energy conversion capability offers a method to effectively scavenge biomechanical energy from the human body itself or the environment. Beyond biomechanical energy generation and biosensing, FENGs’ intrinsic properties enable bidirectional conversion of energy between the electrical and mechanical domains, broadening the scope of their application in bioelectronics. The giant dipoles inside the FENG are reshaped by changes in the charge density on the surface electrodes or the electric field across the thickness, which allows the FENG to act as an actuator when extra charges are transferred to its surface electrodes (or when there is a potential difference between them). One of the represented applications utilizing FENGs’ actuating behavior is production of acoustic waves with a broad range of frequencies (up to megahertz),^37–39^ which stimulates a series of applications in bioengineering.^27,40–42^ While researchers are continuously delving into theoretical analysis, inventive manufacturing methods, novel system designs, enhanced stability, etc., FENGs have already proven their broad prospects as biomedical devices for real-world clinical applications for patients in different countries.^43–45^ FENGs are poised to unleash their potential in the field of bioengineering that complements piezoelectric technologies and, in certain cases, may even serve as a substitute for them.

Because the FENG is a relatively young technology, its name has evolved along with its development, and various groups/scholars frequently make reference to it using different terms. The name “FENG” was only coined recently, but it quickly gained popularity and academic acceptance.^23,34,46–53^ There are two possible explanations for this. The first one is purely based on the history of the term; since the name “ferroelectret” was coined by Bauer et al.^19^ and later published in 2004, ferroelectret has been widely accepted to refer to this technology. Second, the name “FENG” could be interpreted as a homage to “TENG,” a well-known technology with close overall functionality. Over the last decade, FENG research has maintained strong growth momentum in line with the maturity of this technology.

**Figure 1.**
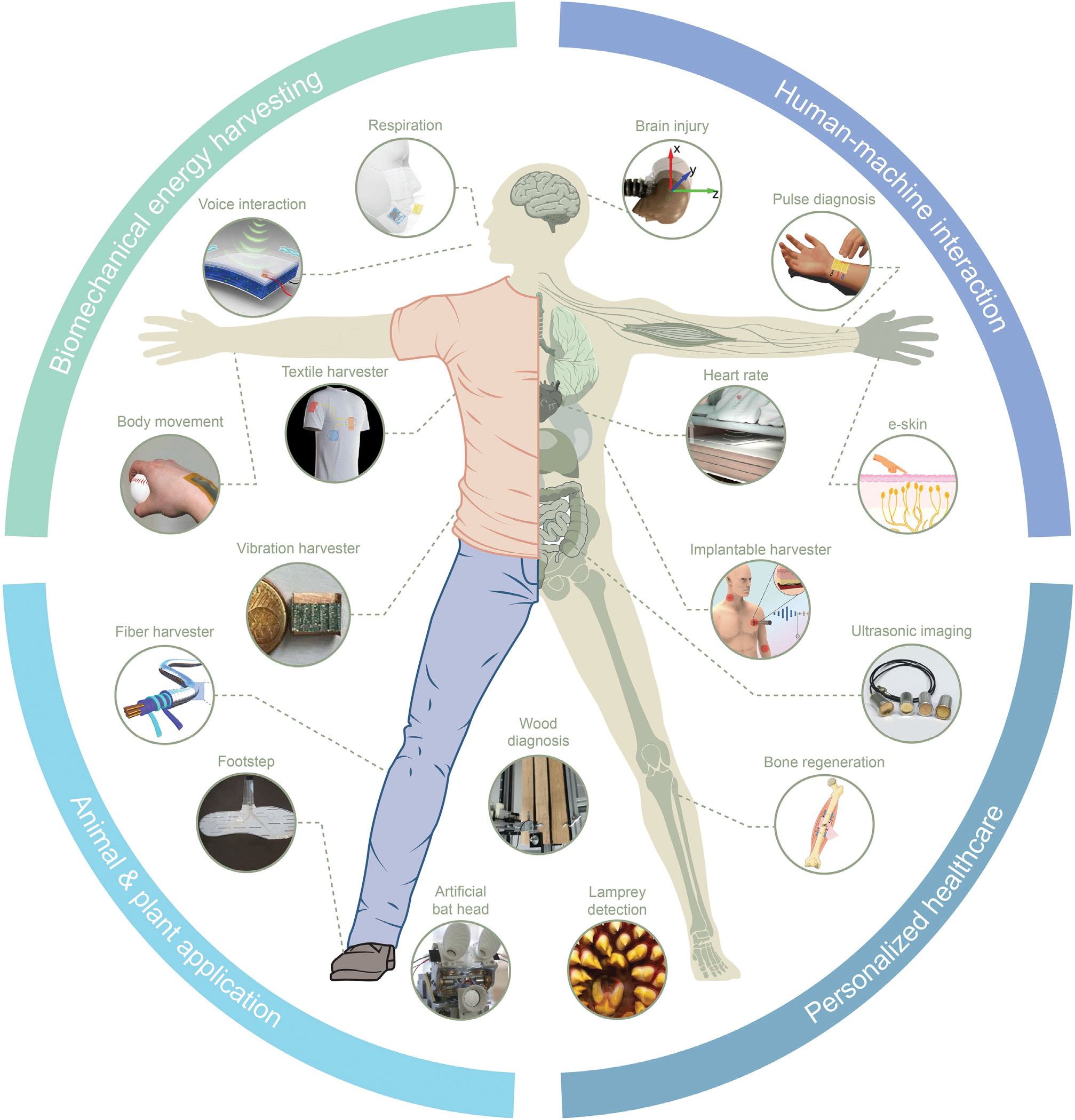
Schematic of the diverse applications of FENGs in bioengineering The diverse applications of FENGs in bioengineering including human-machine interaction, biomechanical energy harvesting, personalized healthcare, and animal and plant applications.

This review provides a retrospective of FENGs’ latest developments for advancing bioengineering from various perspectives involving human-machine interaction, biomechanical energy harvesting, personalized healthcare, and animal and plant applications (Figure 1). First, FENGs’ capability to function as different types of information exchangers between humans and machines during the ongoing information revolution is summarized. Then, the advancement of electricity production from various biomechanical motions using wearable or implantable FENGs as the energy supply is discussed. The applications of FENGs for personalized healthcare concerning the human heart, respiration, blood vessels, brain, bone, pulse, and medical imaging are introduced. Furthermore, the distinctive role that FENGs play in bionic technology, invasive animal detection, and plant protection is demonstrated. Finally, conclusions and viewpoints on the current challenges and potential research directions for FENGs are offered. This review aims to provide a thorough understanding of FENGs’ most recent contributions to the development of bioengineering and to raise awareness of the various applications of this versatile technology to improve the quality of human life.

## HUMAN-MACHINE INTERACTION

With the prospect of a smart and connected society in the not-too-distant future, humans are interacting with machines more frequently to gather information and make data-driven decisions, which should improve quality, efficiency, and productivity.^54–56^ The recent rapid development of AI, IoE, wearable/portable electronics, robotics, virtual reality (VR)/augmented reality (AR), neural interfaces, etc. has further strengthened HMIs’ ability to act as a bridge between humans and machines,^11,57–65^ to the point where the technology is also being considered for security, privacy, dependability, and user convenience applications.^23,66^ FENGs are considered a promising candidate technology to play a special role in promoting HMIs in various ways because of their excellent performance and broad interactive applications in our daily lives. Here, four categories of HMIs are discussed, tactile perception interface,^28,67,68^ voice interactive interface,^23,51,69^ body-movement-monitoring interface,^24,25,70^ and footstep-tracking interface,^71,72^ which encompass multiple fundamental interactions that people experience ubiquitously in their daily routines.

### Tactile perception interface

**Figure 2.**
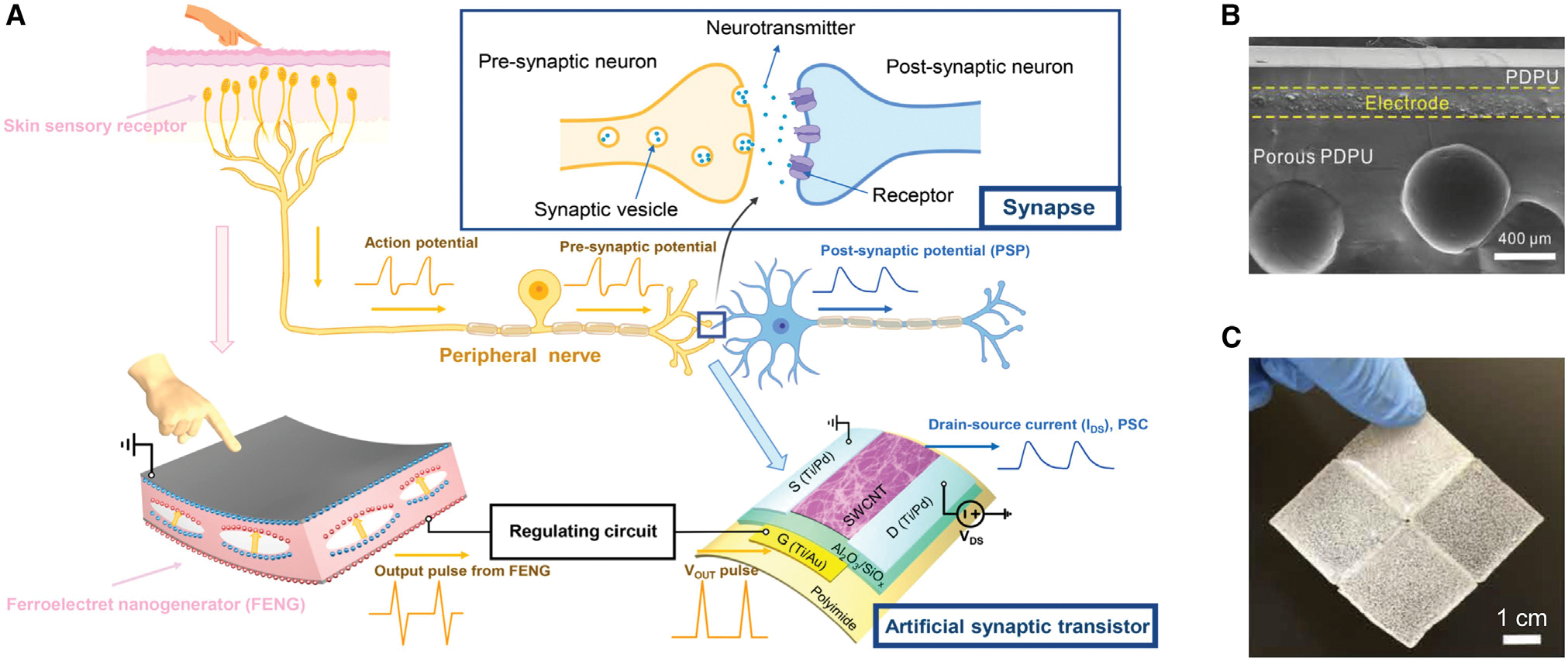
FENGs for voice interaction interface (A) Neurological electronic skin based on a flexible FENG and artificial synaptic transistor. Reproduced with permission.^67^ Copyright 2020, American Chemical Society. (B and C) Porous and self-healing PDPU elastomer as electronic skin for self-powered sensing. (B) Scanning electron microscopy (SEM) image of the cross-section of the device with internal voids. (C) A built-up device that was combined from four separate parts through self-healing. Reproduced with permission.^28^ Copyright 2020, American Association for the Advancement of Science.

Human tactile perception is a complex process related to the detection of pressure and vibration in spatiotemporal mechanical deformations on the glabrous skin.^73^ This research topic requires the integration of tactile sensing capabilities into intelligent robotic systems by equipping sophisticated and delicate sensors to emulate human skin.^74–79^ Recently, FENGs have emerged as a viable candidate for the development of artificial skin. By combining the flexible artificial synaptic transistor and a FENG, Wan et al.^67^ emulated the behavior of a neurological electronic skin (Figure 2A). The authors used a thin-film FENG to imitate the mechanoreceptor and peripheral nerve for transducing and relaying force stimulus information to the synapse. In this artificial sensory skin, the FENG transformed the tactile information (force amplitude and frequency) of the physical contact into presynaptic action potential pulses, which were then passed to the gate of the synaptic transistor to render changes in drain current, simulating the modulation of synaptic weight in a biological synapse. When the FENG was stimulated with forces of 41.3 N or 14.2 N at 0.67 Hz, a total synaptic weight modification of 118.9% or 56.5% for potentiation at 41.3 (±1.2) N or 14.2 (±0.6) N and −72.1% or −44.3% for depression at 41.3 N or 14.2 N was observed. Successfully reproducing analog-like synaptic weight modulation with force-encoded information is a step toward use of flexible electronics for neuroprosthetic applications. Later in 2021, the same group reported an artificial multimodal sensory-memory system that was endowed with bioreceptor-like sensory capability and synaptic-like memory to mimic biological sensory-memory behaviors.^68^ Three of the five basic human sensing abilities (i.e., touch, hearing, and sight) were enabled by utilizing a FENG as the tactile sensor and the acoustic sensor while using a phototransistor as the optical sensor. Another intriguing study was done by Xiong et al.,^28^ who created an adhesive, gas-tight, and self-healing porous elastomer that promises to be a freestanding, wearable, functional tactile skin for self-powered sensing of touch pressure, human motion, or Parkinsonian gait (Figures 2B and 2C). This supramolecular polysiloxane-dimethylglyoxime-based polyurethane (PDPU) porous elastomer generated electricity using the FENG’s operating principle through the interactions between the trapped air and the elastomeric matrix when compressed periodically. This elastomer has the advantage of eliminating the poling process during fabrication, whereas conventional FENGs typically necessitate a strong electric field to break down the gas or air in the void. Apart from good stretchability (1,800%) and high compressive strain (60%), this porous elastomer also showed superior healing efficiency (66.7% for 4 h), making it a good material for self-powered smart skin on soft robots and humans. The proposed FENG-based tactile perceptions in the studies mentioned above have shown the potential for FENG application in soft robots, VR, robot-assisted surgery, prosthetic hands or arms, secure human-robot collaboration, etc.

### Voice interaction interface

**Figure 3.**
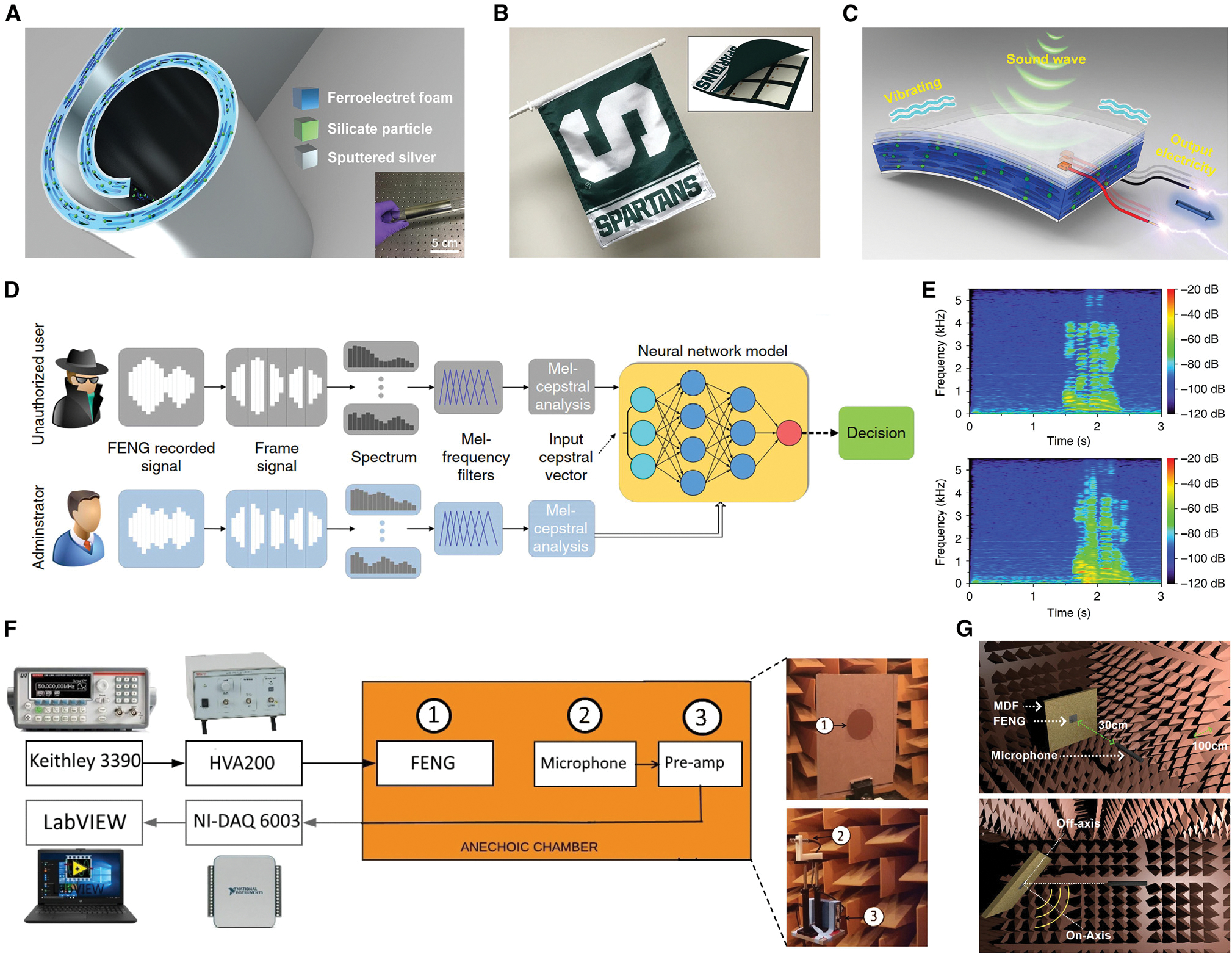
FENGs for voice interaction interface (A–I) FENG-based dual-functional and self-powered thin-film flexible acoustic transducer that operates as a loudspeaker and microphone for flexible electronics. (A) Schematic structure of a large-area flexible FENG-based acoustic device. The inset shows an optical image of the device on a glass tube. (B) FENG-based music-playing flag. Inset: schematic showing FENG patches inside the flag. (C) Mechanism for the FENG-based microphone’s conversion of sound waves into electrical signals. (D) FENG-based voiceprint identity recognition system using artificial neural networks. (E) Recorded spectrogram of voice code spoken by the administrator (top) and an unauthorized user (bottom). Reproduced with permission.^23^ Copyright 2017, Springer Nature Group. (F and G) Performance investigation of the FENG-based loudspeaker. (F) Experimentation in testing with the FENG, the microphone, and the pre-amp inside an anechoic chamber. (G) Schematic of SPL directivity measurement. Reproduced with permission.^51^ Copyright 2020, Elsevier.

Along with tactile sensing, acoustic interaction (i.e., hearing and speaking) is another essential and convenient link between humans and machines.^80,81^ Noteworthy, the acoustic radiation generated by FENGs is strong enough to counteract gravity and levitate particles in mid-air.^82^ Furthermore, the FENG is also capable of harvesting acoustic energy.^83^ Based on the direct and reverse interactions, FENGs can transform the propagated sound wave into digital signals that are understood by machines; conversely, the machines can also convert the digital signals into sound waves that people can hear and comprehend. As shown in Figures 3A–3E, Li et al.^23^ demonstrated a FENG-based dual-functional and self-powered, thin-film, flexible acoustic transducer that operated as a loudspeaker and a microphone for flexible electronics. To demonstrate the applicability and functionality of the device, a FENG-based music-playing flag that can operate under regular wind conditions was created. In addition, the process can be carried out in reverse to produce a FENG-based microphone that transforms sound waves into electrical signals. This FENG device was responsive to sound waves across a wide range of frequencies. Two examples served as demonstrations in the study: a high-fidelity recording of a symphony and a voiceprint identity recognition system that acts as a watchdog for security. According to the user’s requirements, the FENG could switch its functionality between being a loudspeaker or a microphone for HMI. Later in 2020, an investigation into the FENG-based loudspeaker was conducted (Figures 3F and 3G),^51^ which demonstrated the dependencies of sound pressure level (SPL) produced by FENGs in response to AC voltage stimulus, surface area, geometric shape, and multi-layer configurations. Moreover, a theoretical model based on boundary element methods was introduced to relate the FENG’s acoustical and electrical domains. In a different study, experiments carried out in an acoustic anechoic chamber were used to characterize various parameters of interest in FENG-based microphones.^69^ The frequency response for FENGs with different areas was observed to be nearly flat over the entire range of human audibility, and the sensitivity rose linearly with an increase in the area of the FENG. Because of their flexibility, FENG-based microphones can be configured into different shapes, enabling reconfigurable tuning. Driven by the rapidly increasing demand for wearable/portable devices, FENG-based acoustic devices are able to boost the experience of HMI through convenient voice communication and safeguarding sensitive personal data (such as daily GPS position, physical activity, and health status).

### Body-movement-monitoring interface

**Figure 4.**
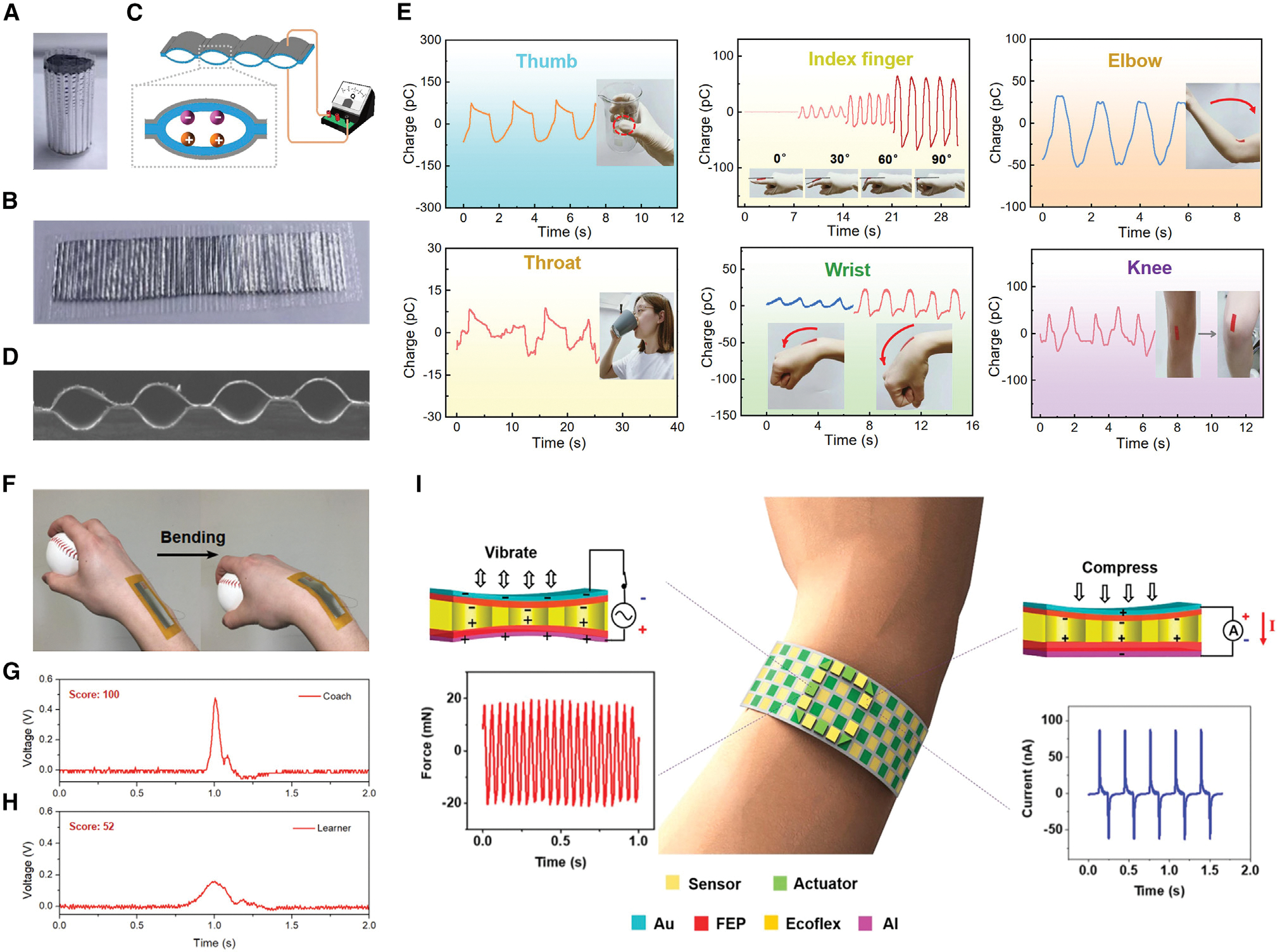
FENG for a body-movement-monitoring interface (A–E) Compressive and stretchable FENG with an air-filled parallel-tunnel structure for self-powered body motion detection. (A and B) Optical images of the fabricated FENG in (A) rolled configuration and (B) planar configuration. (C) Schematic of the device’s sensing unit and charge distribution inside the film void. (D) Cross-sectional SEM image. (E) Demonstration of FENG-based wearable sensors for different types of self-powered body motion tracking. Reproduced with permission.^24^ Copyright 2022, Elsevier. (F–H) Demonstration of FENG application in athletic assessment. (F) Photographs of a baseball player wearing the FENG sensor, bending his wrist while throwing the ball. (G and H) The output voltage signals from (G) the coach and (H) the learner when throwing the baseball. Reproduced with permission.^70^ Copyright 2019, IEEE. (I) FENG-based actuator/sensor array as a wearable device for real-time feedback and long-distance haptic communications. Reproduced with permission.^25^ Copyright 2019, American Chemical Society.

Adaptable, light, comfortable, and self-powered wearable sensors that can track human motion are in high demand because they could provide real-time health status data and track human motion for extended periods of time to create a database.^24,28,84–88^ Biomechanical movement can be transformed into electrical signals for information processing and decision-making through motion-tracking interfaces.^57^ As a useful technology in this application scenario, FENGs have been explored in various ways to either be directly attached to or worn on the human body for movement monitoring.^24,25,35,36,70,89–91^ Because of its thin-film flexible structure, high sensitivity, self-powered feature, and compatibility with flat or uneven substrates, a FENG can detect signals from various mechanical motions, including compressing, stretching, rubbing, twisting, and folding. In 2022, Ma et al.^24^ demonstrated a self-powered electronic skin that can not only detect the bending motions of fingers, wrists, elbows, or knees but can also measure gripping force (Figures 4A–4E). These versatile motion detectors were built based on a FENG with an air-filled parallel-tunnel structure that was compressible and stretchable. Attributed to the tensile properties of the parallel-tunnel structure and the good charge storage stability of the FENG, these laminated films exhibit a high longitudinal piezoelectric coefficient (5,100 pC/N), wide pressure detection range (0.03–62 kPa), and sensitive transverse piezoelectric response (tensile sensitivity of 1,600 pC/mm). Assisted by machine learning, this detector enabled intelligent gesture recognition that can quantify wrist movement activities, distinguish between different finger bending angles, and record laryngeal vibrations caused by swallowing. Another study aiming to improve athletes’ motion training presented a self-powered, water-resistant bending sensor based on the transverse piezoelectric effect of FENGs (Figures 4F–4H).^70^ The device was able to extract information regarding the bending curvature and speed of an athlete’s wrist during sports like baseball or basketball. In addition to sensing biomechanical motion, integration of perception and action in response to environmental or human stimuli is important for HMIs, where the functions of sensing and actuation are inextricably linked. Zhong et al.^25^ presented a FENG-based flexible actuator/sensor array for interactive feedback communications with the features of high resolution, light weight, and low manufacturing cost (Figure 4I). The functionality of the actuator can be realized by using an electrostatic force to induce vibrational stimulation that can provide haptic feedback to human skin. In this approach, incorporation of actuators and sensors into a wearable device enables real-time sensing-actuating feedback as well as long-distance haptic communications. It can be expected that human body movement monitoring will keep benefitting the development of personal wellness monitoring, sports training, humanoid robots, and haptic feedback for VR.

### Footstep-tracking interface

**Figure 5.**
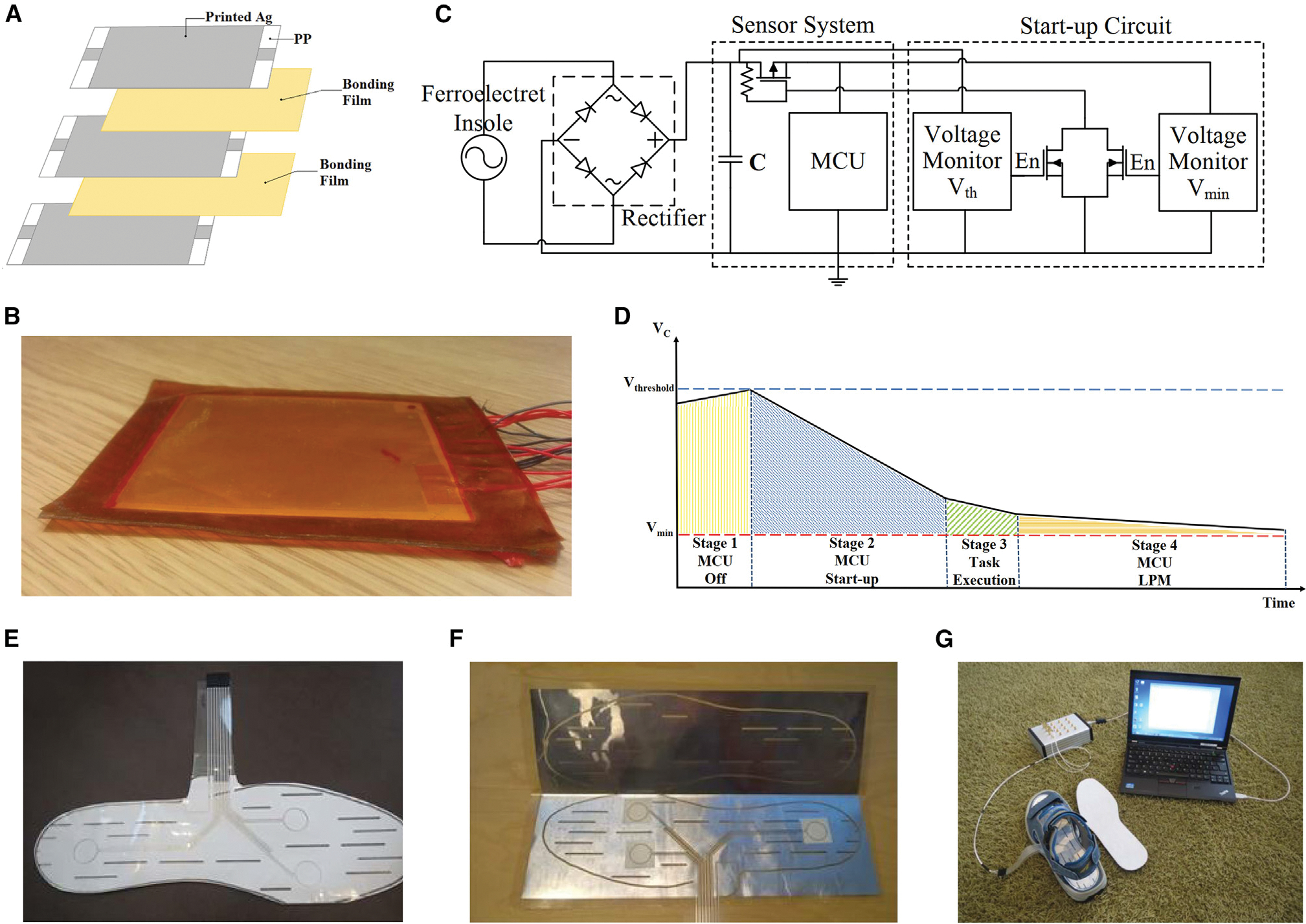
FENG for footstep-tracking interface (A–D) Intermittently powered energy-harvesting footstep tracker based on a FENG that eliminated the energy storage component. (A) Schematic of the multi-layer structure of the FENG insole. (B) Optical image of a 30-layer FENG insole. (C) Circuit diagram of the intermittently powered FENG footstep tracker. (D) Process of counting a step that was divided into four stages. Reproduced with permission.^71^ Copyright 2017, IEEE. (E–G) FENG in-shoe sensor for measuring plantar pressure distribution to early identify and prevent individuals’ risk of ulceration. (E) Optical images of the overall appearance of the in-shoe sensor. (F) Optical image showing the construction of the in-shoe sensor. (G) Plantar pressure distribution test measurements, where the in-shoe sensor was located inside a shoe and covered with an insole. Reproduced withpermission.^72^ Copyright 2018, IEEE.

Footstep tracking is one of the most popular fitness tracking strategies and is widely adopted by commercial fitness trackers and smartphones. The global market size for fitness trackers is estimated to reach $92 billion by 2027.^92^ For fitness tracking, Rodriguez et al.^71^ proposed an intermittently powered, energy-harvesting footstep tracker based on a FENG that eliminated the energy storage component (Figures 5A–5D). This device sustained its operation by collecting energy from footsteps using a FENG-based insole that produced electricity when subjected to mechanical stress. A microcontroller unit (MCU) with a low-power, non-volatile memory awakened at each step during the fitness tracking monitoring and retained data during the power outages between steps. The results show that this self-powered step counter has a step-counting error of less than 4% when walking, which is much better than using up-to-date smartphone fitness applications. In addition, accurately monitoring human gait is critical for health evaluation and early diagnosis. The presence of gait abnormalities could be a predictor of the risk of developing diseases.^93^ For example, plantar force measurements have revealed a link between excessive mechanical stress and foot ulceration.^94^ Rajala et al.^72^ developed three in-shoe sensors for measuring plantar pressure distribution based on three film-type sensor materials: PVDF, cellulose nanofibrils (CNF), and EMFi (ElectroMechanical Film, a commercially available FENG material) to early identify and prevent individuals’ risk of ulceration (Figures 5E–5G). Three sensor channels made up the insole electrode sheet: two for the lateral and medial metatarsal heads and one for the heel. According to the sensitivity measurements, the 0.25-mm-thick FENG-based in-shoe sensor had the highest sensitivity for all three measured locations. In contrast to PVDF and CNF in-shoe sensors, which had sensitivity values of 15.9 ± 2.7 pC/N and 1.1 ± 0.3 pC/N, respectively, the EMFi in-shoe sensor’s lateral metatarsal head sensitivity reached 166.0 ± 4.6 pC/N. Positive results for using the FENG-based in-shoe sensor to prevent pressure ulcers in humans have been found in testing and comparison. Utilizing FENGs as a footstep-tracking interface not only paves the way for real-time and long-term gait monitoring but also presents an avenue for remote clinical biomotion analysis.

## BIOMECHANICAL ENERGY HARVESTING

For regular operation and communication of wearable electronics, biomedical devices, sensor networks, etc., a sustainable and dependable power source is essential.^2,11,55,95–97^ Traditional battery-based power supply systems have limitations when supplying sustainable energy to biomedical devices throughout or inside the human body; sometimes replacing batteries is a complicated or even impossible task. Moreover, batteries face challenges such as limited lifespan, hazardous chemicals, large size, and heat production that may hinder their widespread use in biomedical devices.^98^ The human body has constant and widespread biomechanical energy, displayed in daily actions like walking,^99^ limb movements,^100^ typing on a keyboard,^66^ or through biological processes,^101–103^ that are often unnoticed. Electronics with low power consumption can use FENG devices/systems to transform various forms of biomechanical energy into electricity, giving them a supply of renewable energy.^52,53,104–106^

### Pressure bioenergy harvesting

**Figure 6.**
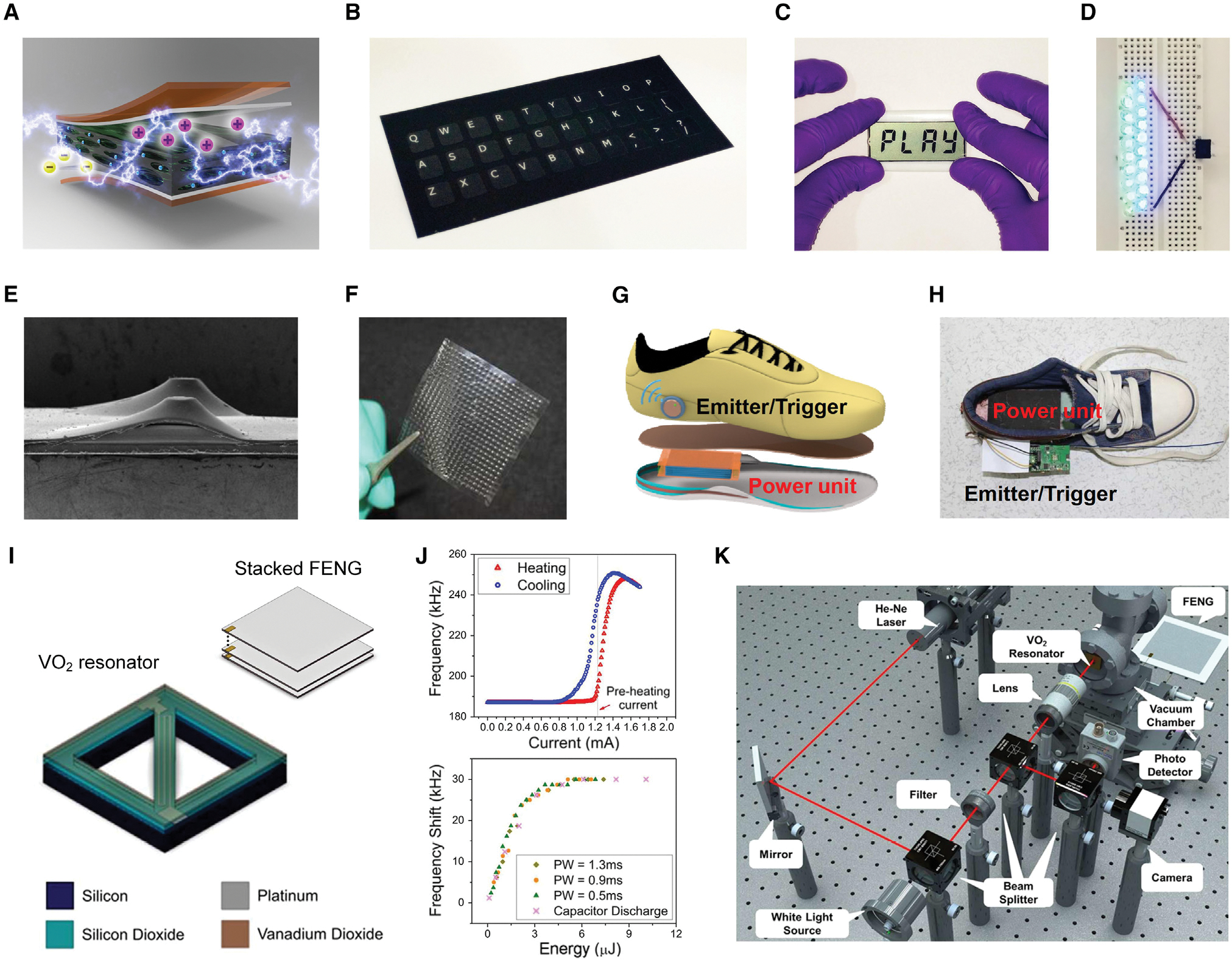
FENG for pressure bioenergy harvesting (A–D) Flexible PP-based FENG for applications of self-powered electronics. (A) Exploded view of the encapsulated biocompatible FENG. (B) Photograph of a FENG-based thin-film flexible/foldable self-powered keyboard. (C) A self-powered LCD touchscreen that can scavenge energy from finger touch and display the word “PLAY.” (D) 20 LEDs lit by a FENG through a one-time hand press. Reproduced with permission.^34^ Copyright 2016, Elsevier. (E–H) Laminated EVA/BOPP FENG for energy harvesting from walking motion. (E and F) Cross-sectional SEM image (E) and photograph (F) of a laminated FENG. (G and H) Schematic (G) and photograph (H) of the self-powered and self-triggered wireless emitting system integrated with a shoe. Reproduced with permission.^107^ Copyright 2016, Royal Society of Chemistry. (I–K) Impact-activated programming of electro-mechanical resonators through a FENG and VO_2_. (I) Schematic of a VO_2_-based MEMS resonator and the stacked FENG. (J) Hysteretic major resonant frequency loop for bridge structure as a function of the current (top part) and frequency shift as a function of energy delivered by the pulse (bottom part). (K) Schematic of the optical measurement setup built to verify the frequency shift of the resonator through the FENG. Reproduced with permission.^50^ Copyright 2018, Elsevier.

The most fundamental working mode for a FENG is conversion of dynamic pressure into electricity. Generation of pressure is ubiquitous when people physically interact with objects or the environment. A typical application of a FENG in human body energy harvesting for electronic devices is self-powered touchscreens, which could be used in smartphones, tablets, E-ink papers, or touchscreen panels. Li et al.^34^ demonstrated integration of a polypropylene (PP) FENG device with a 4-bit LCD screen as a step toward such implementation (Figures 6A and 6B). The electrical energy harvested from biomechanical energy via pressure from the users’ hands enabled letters to be displayed on the screen. Because a FENG device is thin and lightweight, it could be easily integrated into a range of small electronic devices, providing an environmentally friendly alternative to traditional charging methods by utilizing the user’s operational process. Additionally, by converting the mechanical stimuli that were applied to the keyboard into an electric signal when a key was pressed, Li et al.^34^ created a flexible, foldable, self-powered keyboard that can be folded down for portability (Figure 6C). This presents a means of transforming the current large-volume, battery-operated keyboards into portable, powerless gadgets that can be folded down to the size of a business card and used as an add-on for smartphones or tablets. Moreover, a stacked FENG is able to harvest bioenergy from hand presses for powering commercial LED lights (Figure 6D). Aside from hands or fingers, FENGs can be embedded in shoes to harvest energy induced by walking pressure. For instance, Zhong et al.^107^ developed a laminated FENG based on ethylene-vinyl acetate (EVA) and biaxially oriented PP (BOPP) that can efficiently harvest energy from walking in harsh environments (Figures 6E–6H). Using the hot-pressing technique, a cellular structure with regular air bubbles was created. The charges on the EVA and BOPP exterior surfaces of the film self-recovered after the film was soaked in water and then naturally dried. To efficiently capture energy from walking motion, a power unit composed of five flexible FENGs with sandwich structures was embedded into shoes. It was demonstrated that the accumulated energy from human feet collected by this device was capable of supplying power to transmit signals for a wireless radio frequency (RF). Similar foot pressure-powered operation through a FENG is possible with other prevalent communication systems, such as Bluetooth.^108^ Furthermore, micro-electro-mechanical system (MEMS) resonators^109–111^ are widely used in timing reference and wireless communication, and those with multiple-frequency outputs are desired for multi-band reconfigurable wireless comunication systems.^112^ Cao et al.^50^ reported their solution on the FENG device and demonstrated that biomechanical energy can be used to provide the necessary actuation for tuning or programming frequency states in the MEMS structures (Figures 6I–6K).^50^ This was achieved by combining the FENG device with the tuning ability of vanadium dioxide (VO_2_) thin-film coatings to create a MEMS resonator structure that was activated biomechanically. The biomechanical energy obtained through the FENG triggered temperature-induced phase transition of VO_2_, which led to tuning and programming actions. The results indicated that the maximum tuning frequency range for a 300-μm-long VO_2_-based resonator in the bridge structure was about 40 kHz.

### Vibration bioenergy harvesting

**Figure 7.**
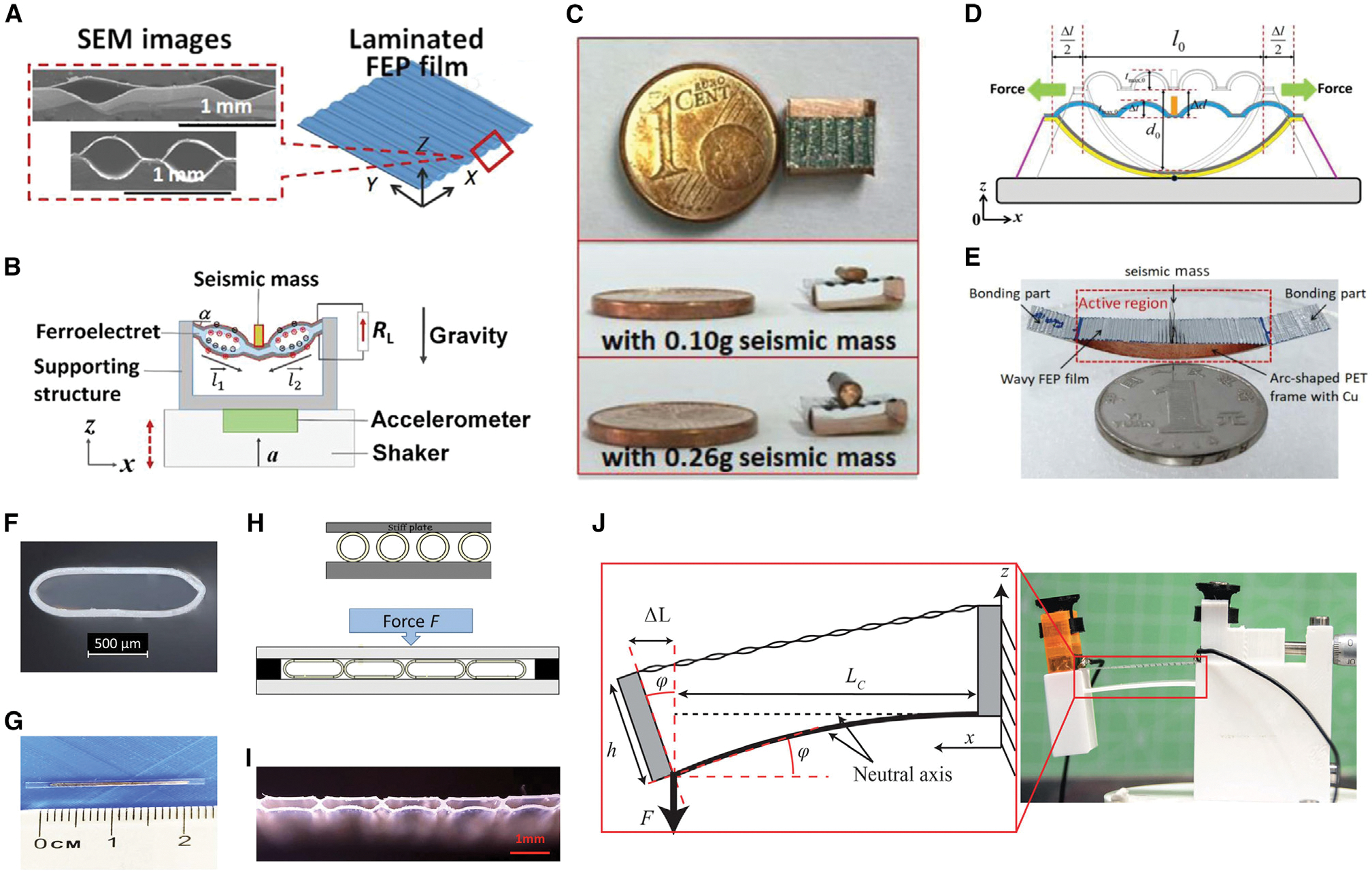
FENG for vibration bioenergy harvesting (A–D) Vibration energy harvester based on a FENG with very large low-frequency transverse piezoelectric activity. (A) Schematic and cross-sectional SEM images of the laminated parallel-tunnel FENG film. (B) Schematic of the experimental setup. (C) Optical images of the FENGs. Reproduced with permission.^49^ Copyright 2018, Elsevier. (D and E) FENG-based vibration energy harvester featuring tunable resonance frequencies. (D) Schematic showing the adjustment of the structural parameters of the harvester. (E) Optical image of the harvester with a seismic mass of 0.06 g cemented in the center. Reproduced with permission.^116^ Copyright 2021, Wiley. (F and G) Compact tubular FENG-based vibrational energy harvester. (F) Cross-sectional micrograph of the stadium-like structure fabricated from FEP tubes with a diameter of 1 mm and wall thickness of 50 μm. (G) Dimensions of the fabricated tube harvester. Reproduced with permission.^117^ Copyright 2020, Wiley. (H and I) Tubular FEP array for energy harvesting. (H) Schematic of the tubular array in its initial configuration and compressed between two heated metal plates. (I) Optical image of the well-ordered tubular assembly under compression. Reproduced with permission.^118^ Copyright 2018, Institute of Physics. (J) FENG-based energy harvesting with a 3D-printed, air-spaced cantilever design. Reproduced with permission.^122^ Copyright 2022, Wiley.

Energy harvested from the vibration of the device induced by human body movement can be readily converted into usable electrical energy and thus provides a sustainable energy source for low-power consumption devices used in wearable electronics, autonomous systems, and remote communications.^113–115^ A vibration bioenergy harvester consisting of a FENG based on fluorocarbon polymers with a large low-frequency transverse piezoelectric coefficient (*g*_31_ = 3.0 Vm/N) was presented by Zhang et al.^49^ (Figures 7A–7C). This coefficient is significantly higher than the corresponding values for PVDF (~0.33 Vm/N) and PZT (~0.02 Vm/N). The FENG device was prepared in a parallel-tunnel form, which was a laminated structure consisting of fluorinated ethylene propylene (FEP) films (12.5 μm thick) fused at elevated temperatures. The authors applied the developed FENG to reduce the energy harvester system’s footprint to dimensions as low as 3 × 5 × 8 mm^3^. At an acceleration of 9.81 m/s^2^, the energy harvester’s power outputs reached 57 μW and 109 μW for seismic masses of 0.09 g and 0.3 g, respectively. Because the results were achieved by utilizing soft materials that were compact, light, and flexible, it may enable use of such devices for supplying power to small gadgets like body-worn electronics and medical implants. Another FENG-based vibration energy harvester featuring tunable resonance frequencies, small size, light weight, and high output power was designed by Ma et al.^116^ (Figures 7D and 7E). By stretching the length of the FEP film loaded with a specified seismic mass of 0.06 g, the resonance frequency of this energy harvester with dimensions of 30 × 10 × 9 mm^3^ can be tuned from 18–70 Hz. It is feasible to tune the resonance frequency by changing the width of the structure or the seismic mass as well as varying the length of the wavy FEP film. In addition, compact, tubular, FENG-based, vibrational energy harvesters were presented by Zhukov et al.^117,118^ (Figures 7F–7I). By mechanically deforming the FEP tubes at an elevated temperature (250°C), the designed tubular vibrational energy harvesters can be fabricated from low-cost FEP tubes. A bias voltage of up to 6 kV was applied for a brief period to charge the tubes. It was demonstrated that the proposed device can achieve a high dynamic response (~300 pC/N) and output power of up to 300 μW using a seismic mass of 80 g at the resonance frequency. Recent studies also indicate that 3D printing can be utilized to create smart devices with electro-mechanical responses, including FENGs.^119–121^ In 2022, a FENG vibrational energy harvester was proposed using a 3D-printed air-spaced cantilever design (Figure 7J).^122^ The FENG used in this study was 10 × 40 mm^2^ in size and was made of FEP. It exhibited piezoelectric *g*_31_ coefficients of 1.9 Vm/N for static operation and 0.8 Vm/N for dynamic operation. The air-spaced cantilever arrangement used in the energy harvester was created by 3D printing, and the cantilever structural material was polylactide acid (PLA) with a 15% infill and a gyroid fill pattern. Such a cantilever-based vibrational energy harvester can produce a normalized output power of more than 1,000 μW at a resonance frequency of about 35 Hz for a seismic mass of 3.5 g and an acceleration of 0.98 m/s^2^. The demonstrated energy harvester was effective at low acceleration amplitudes, making it well-suited for the capture of biomechanical vibrational energy in daily life.

### Textile-based bioenergy harvesting

**Figure 8.**
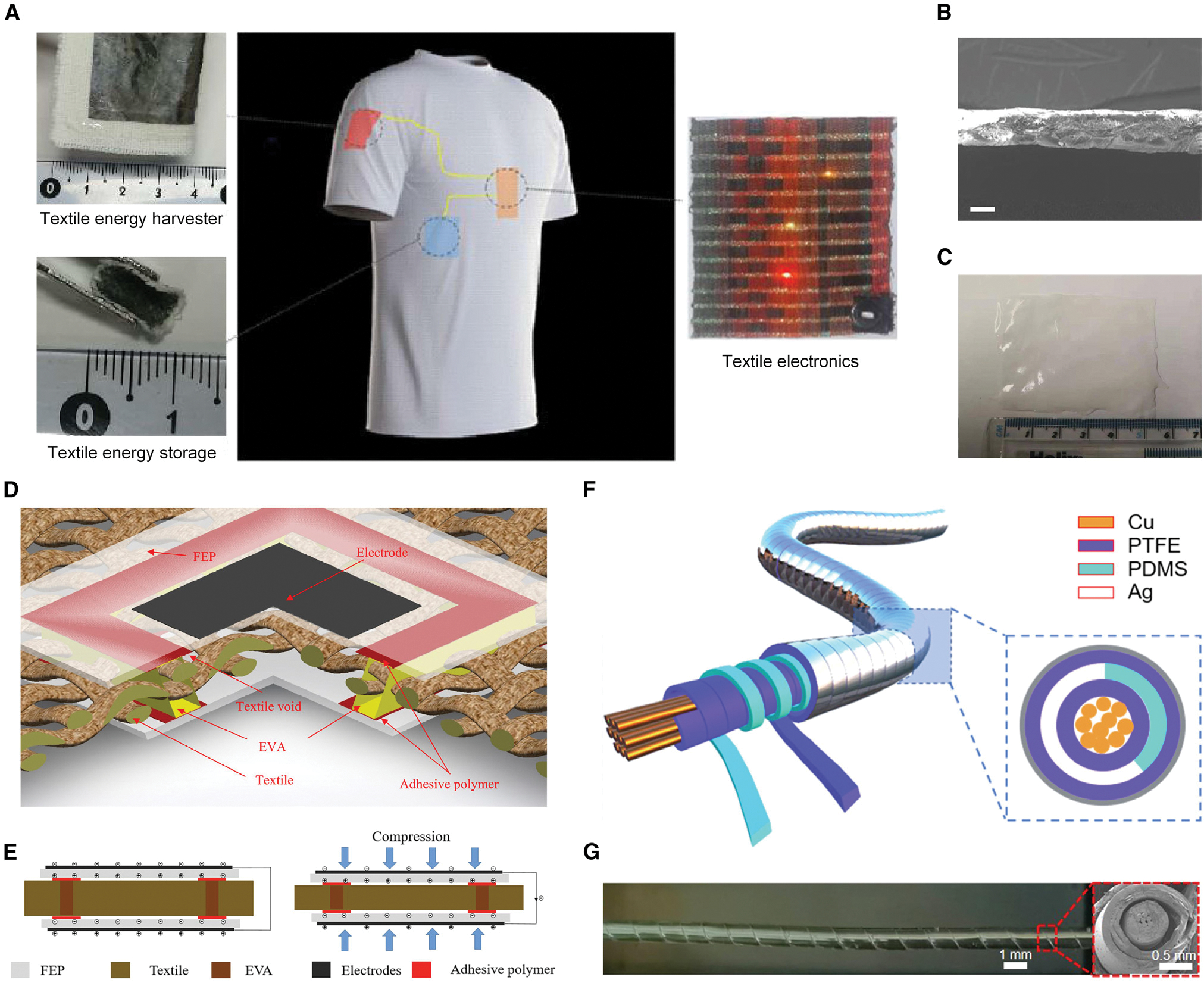
FENG for textile-based bioenergy harvesting (A) Schematic of the textile power module, comprising a spray-deposited supercapacitor, FENG energy harvester, and electronics all integrated into the same single layer of textile. Reproduced with permission.^124^ Copyright 2019, Wiley. (B and C) PDMS-ZnO textile-based FENG. (B) Cross-sectional image (B; scale bar, 100 μm) and optical image (C) of the textile-based FENG. Reproduced with permission.^125^ Copyright 2019, IEEE. (D and E) A textile-based FENG with the lamination of two thin FEP films onto the back and front surfaces of a conventional textile. (D) Schematic of the structure (D) and working principle (E) of the FEP-textile FENG. Reproduced with permission.^126^ Copyright 2022, Institute of Physics. (F and G) Fiber-based FENG with a semi-supported core-shell structure. (F) Schematic (F) and optical image (G) of the fiber-based FENG. Reproduced with permission.^26^ Copyright 2021, American Chemical Society.

Human civilization has needed and used textiles for thousands of years. The integration of textiles with energy harvesters is expected to offer a wearable, sustainable, and eco-friendly energy solution for bioenergy harvesting.^123^ The rapid development of electronic textiles creates a need for power supplies that are compatible with the mechanical properties of the textiles. A textile power module that combines a FENG-based biomechanical energy harvester and solid-state supercapacitor energy storage was presented by Yong et al.^124^ (Figure 8A). It was fabricated in a single layer of woven cotton fabric using a combination of standard processes employed in the textile industry, such as screen printing or spray coating. The FENG harvester captured energy from human body movement, while the power management circuit converted the electrical energy into a form suitable for storing in a supercapacitor, which served as an energy reservoir. In this work, a textile-based FENG was formed from the combination of FEP and cotton, where the textile acted as a spacer between the FEP films. After poling, the FEP textile FENG’s piezoelectric coefficient *d*_33_ was measured to be 520 pC/N. The textile power module was very flexible, and the FEP-based FENG can produce electric energy from a compressive force of 350 N across a 70 MΩ resistive load with an instantaneous output voltage of ~10 V and a power density of ~0.86 μW/cm^2^. Additionally, Shi and Beeby^125^ presented a low-cost, stretchable textile FENG made of a nanoparticle mixture of polydimethylsiloxane (PDMS) and ZnO that was spin cast on both sides of a textile (Figures 8B and 8C). In this case, the textile filled the space left by the void in the conventional FENG structure, and the composite of PDMS and ZnO served as a charge carrier material. According to the experimental results, the piezoelectricity of the PDMS-ZnO textile FENG reached 380 pC/N. In 2022, Shi and Beeby^126^ reported a textile-based FENG with lamination of two thin FEP films onto the back and front surfaces of a conventional textile, forming a sandwich structure to harvest energy from the human body (Figures 8D and 8E). The authors showed that the fabricated FEP textile FENG can exhibit increased piezoelectric properties with thinner textiles, and the highest measured stable maximum piezoelectric coefficient d_33_ was 987 pC/N. In their demonstration, the proposed FENG based on FEP-silk textile was able to generate a peak output power density of 2.26 μW/cm^2^, and its output performance had hardly changed when applying cyclical 30-kPa pressure and 90 bending 60,000 times. This technique offers a straightforward and effective method for powering wearable electronic systems through modification of standard textiles. In addition, a fiber-based FENG with a semi-supported core-shell structure that features good reliability, stability, and universality was recently developed (Figures 8F and 8G).^26^ This FENG consisted of an inner copper electrode, an inner electret layer of PDMS, an outer electret layer (polytetrafluoroethylene [PTFE]), and an outer silver electrode. A PDMS strap (100 μm thick and 1 mm wide) was twined on the commercial PTFE-insulated copper wire. PDMS served as a supporting layer, and metalized PTFE was tightly twined on the fiber with the metalized surface outside. The entire FENG fiber was encapsulated by PDMS through dip coating to improve its reliability. Experiments revealed that the FENG fiber had the capacity to produce 40-V voltage or 0.6-mA current, and no obvious degradation of performance under long-time continuous work (>16 h) and different humidity environments (20%–95%) was observed. With these advantages, FENG fibers showed their potential for the concept of self-powered textiles.^127^

### Implantable ultrasonic energy harvesting

**Figure 9.**
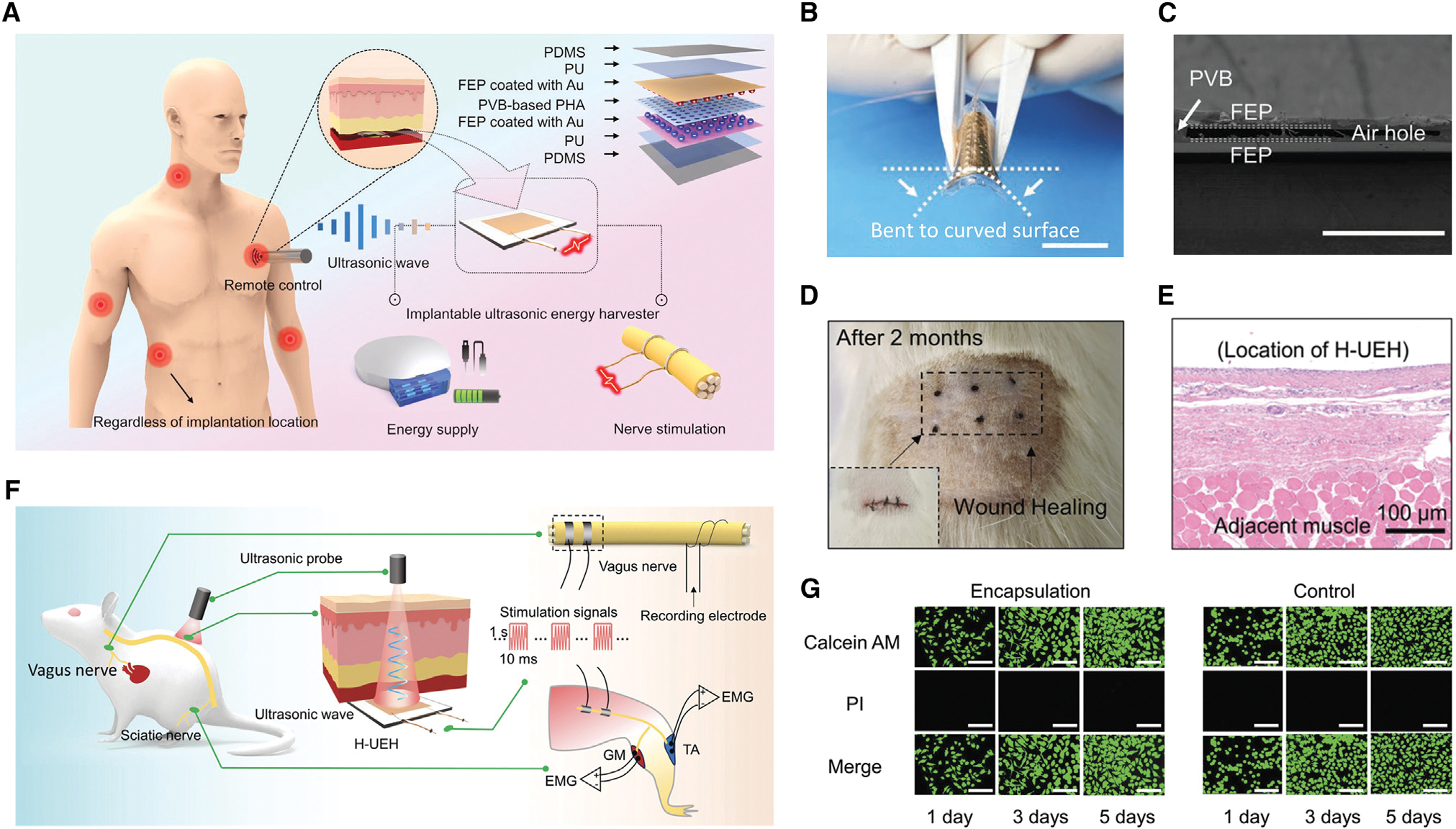
FENG for implantable ultrasonic energy harvesting (A) Schematic of H-UEH as implantable bioelectronics for energy supply and neuroprosthetics and structure diagram of H-UEH. The device can be driven by ultrasound remotely to produce tunable electrical outputs. (B) Optical images of the device when it is bent to a curved surface (scale bar, 1 cm). (C) SEM image of the sandwich structure (scale bar, 500 μm). (D) Optical image of wound healing 2 months after surgery. The inset shows an optical image of the wound just after the implantation surgery. (E) Hematoxylin and eosin (H&E)-stained images of tissue sections after 56 days of implantation. (F) Schematic of the H-UEH driven by ultrasound for stimulating peripheral nerves. (G) Live/dead staining assays of healthy mouse fibroblasts (L929) cultured on PDMS-PU-encapsulated H-UEH after 1, 3, and 5 days (scale bar, 100 μm). Reproduced with permission.^128^ Copyright 2022, Wiley.

Implantable medical devices may require more sustainable power through energy harvesting than wearable electronics. In general, current batteries have two drawbacks that are apparent for *in vivo* applications: first, they generate heat (which makes the systems less efficient), and they could involve toxic substances; second, a sudden shut-down of the system can put the patient’s life at risk, not to mention that the need to replace the batteries can result in emotional distress for the patient.^98^ In light of this, wireless energy transmission for implantable medical applications is highly desired and has the potential to be used in cutting-edge medical protocols for neuroprosthetics, wireless power, biosensors, etc. Contrary to *in vivo* energy derived from organ movement, wireless energy can offer tunable transmission power independent of organ shape, implantation site, and body size. In 2022, Wan et al.^128^ presented a multilayered FENG with strain-enhanced piezoelectricity by introducing a parallel-connected air hole array in an interdielectric layer sandwiched between a pair of electrets for a high-efficiency ultrasonic energy harvester (H-UEH), as shown in Figure 9. When implanted into tissues at a depth of 5–10 mm under an ultrasonic probe setup at 25 mW/cm^2^, this device offered a peak output power of about 13.13 mW and a short-circuit current of about 2.2 mA, which was higher than the necessary power threshold of bioelectronic devices and the current threshold of nerve stimulation. The feasibility of the H-UEH-based implantable nerve stimulator was determined by conducting peripheral nerve stimulation (vagus nerve and sciatic nerve) on rat models. A cell culture experiment was conducted to verify the biocompatibility of the FENG ultrasonic energy harvester. The device was additionally subcutaneously implanted into the back of the rats to examine its stability and biocompatibility over an extended period. The wound on the back of the rats completely recovered after 56 days of implant experiments, leaving only a faint scar. This study offers proof of the biocompatibility and stability of the FENG-based ultrasonic energy harvesting technology, indicating an appealing prospect for wireless power supply, drug delivery, or neuroprosthetics in implantable bioelectronics.

## PERSONALIZED HEALTHCARE

Personalized healthcare uses state-of-the-art information technologies, such as IoT, Big Data, cloud computing, and AI, to transform the traditional medical system in an all-around way, making healthcare more efficient, convenient, and personalized.^130–133^ The ability to continuously monitor and improve people’s health through self-care is an important prerequisite for providing optimal personalized healthcare.^2^ With their broad healthcare applications, FENGs can be considered a useful technology to help address the challenge to the existing medical and healthcare services rendered by the sharp increase in the world’s overall population (especially the elderly, a population sector with high demand for medical resources) and the imbalanced development between rural and urban areas. Recently, FENG-based technologies have been used successfully in smart healthcare applications such as heart and respiration monitoring,^43,44^ Alzheimer’s disease prediction,^134^ blood pressure recording,^135^ respiratory infection control,^129^ concussion prediction,^136^ bone injury healing,^137^ pulse diagnosis,^138,139^ and medical imaging via ultrasound.^27,140^

### Heart and respiration

**Figure 10.**
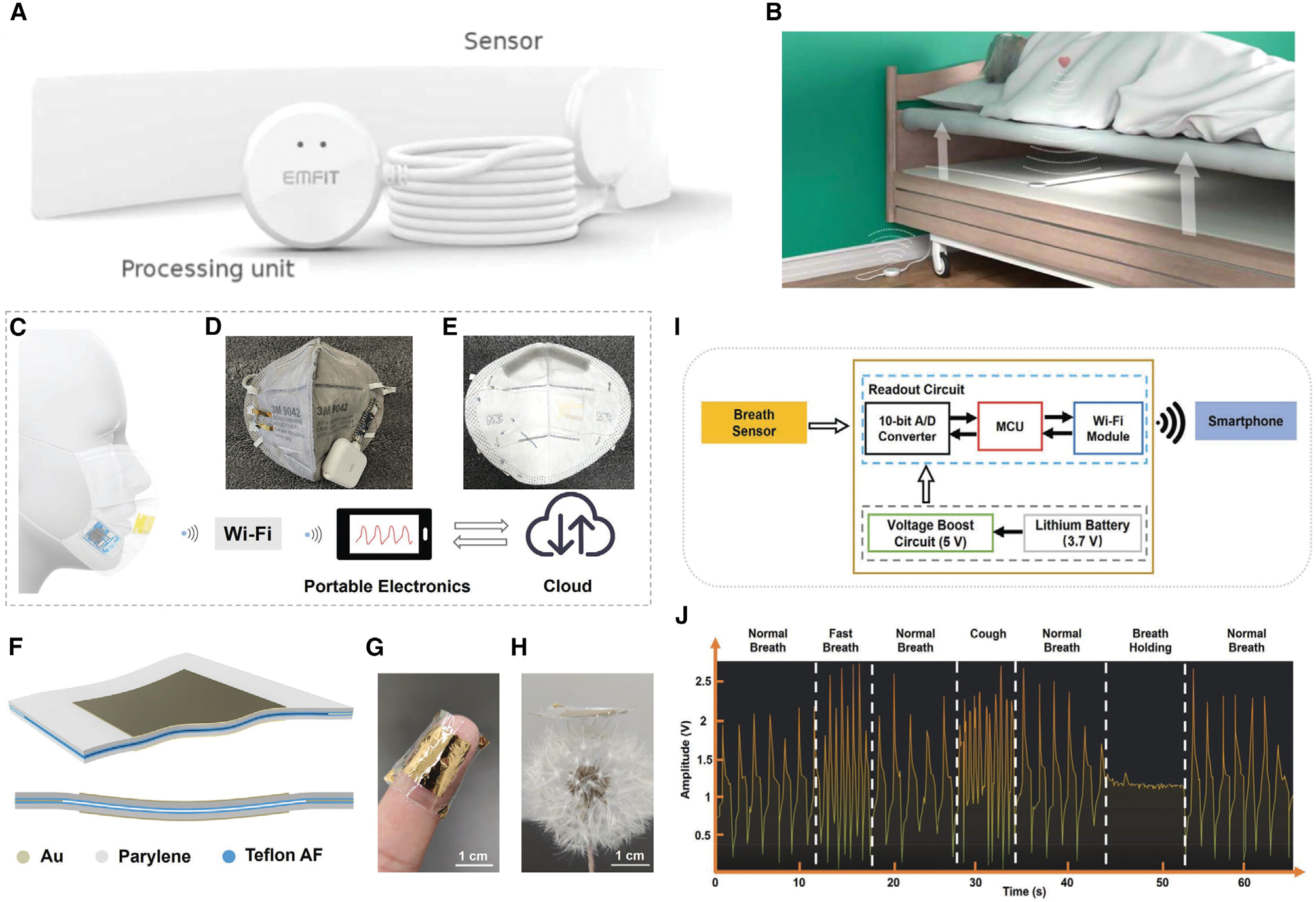
FENG-based personalized healthcare for heart and respiration monitoring (A and B) FENG-based product EMFIT QS for HR and RR monitoring. (A) An unobtrusive sleep monitoring device made by the manufacturer. (B) Schematic showing the FENG sensor placed under a mattress and the processing unit streaming the data to the cloud through the internet.Reproduced with permission.^43^ Copyright 2019, Institute of Physics. (C–J) Smart face mask based on an ultrathin pressure sensor for wireless monitoring of breath conditions. (C) Schematic of the smart face mask for wireless breath monitoring. (D and E) Photographs of the front (D) and back (E) sides of the smart face mask. (F) Schematics of the left and side views of the ultrathin self-powered pressure sensor. (G) Photograph of the sensor surrounding a finger depicting its super flexibility. (H) Photograph of the sensor on a dandelion, which shows its super lightness. (I) Block diagram of the wireless breath monitoring system. (J) Measured continuous and successive breath conditions. Reproduced with permission.^129^ Copyright 2022, Wiley.

A favorable way to monitor vital signs without causing discomfort or disruption of a person’s daily activities is through contact-free, unobtrusive physiological measurements. Non-contact/non-invasive testing lowers the barriers to long-term monitoring by eliminating the need to acclimatize oneself to wearing sensors, as opposed to traditional cardiorespiratory monitoring using skin electrodes, wearable chest belts, or nasal cannulas. For ballistocardiography (BCG) measurement, Ranta et al. ^43^ developed a contact-free thin sleep-monitoring device based on a thin-film FENG that can be tucked beneath a mattress (Figures 10A and 10B). This FENG device, which was named Quantified Sleep (QS), worked with a cloud-based analysis platform. In addition to measuring BCG, respiration, and gross body movements, the QS also calculated heart rate (HR) and respiration rate (RR). The system offers an HR variability analysis and provides a summary of the activities from the previous night, including an estimation of the sleep stage and accompanying sleep quality indicators. During a clinical sleep study in Pirkanmaa Hospital District in Tampere, Finland, a dataset of 34 patients was gathered. The results showed that the HR and RR measurements highly agreed with the references. Using this type of accurate and contact-free FENG device, the subject’s vital signs can be conveniently monitored without discomfort. Aside from unobtrusive physiological measurements for patients, athletes and sedentary people use HR variability (HRV) and HR measurements to monitor their stress and recovery levels. In 2020, Vesterinen et al.^44^ evaluated the precision of QS in measuring HR and HRV during sleep, using an electrocardiogram (ECG)-based device as a reference. Twenty healthy participants from Finland were recruited for the study, and nocturnal recordings of their HR, HRV, breathing, and other body movements were captured using a QS with a size of 6 × 55 cm^2^ that was positioned beneath a mattress. Immediately after the participants went to sleep, the QS measurement began automatically and ended when they got out of bed in the morning. Shortly after waking up, a smartphone, tablet, or computer can access the results as the data are transmitted to the internet. Moreover, FENG devices can play a role in spotting changes associated with the early signs of cognitive impairment in people who are progressing toward Alzheimer’s disease (AD). A prospective cohort study, called Prospective Imaging Study of Aging: Genes, Brain, and Behavior (PISA), was carried out in Australia in 2021 by Lupton et al.^45^ to characterize the phenotype and natural history of healthy adult Australians at high future risk of AD. 137 onsite PISA participants received the QS device for recording continuous resting HR and RR, sleep stage estimates, sleep duration, sleep latency, waking rate, etc. The smart sensing stream was utilized to understand changes in sleep patterns throughout the AD spectrum, from the preclinical stage to late-onset AD, and to examine the impact of disrupted sleep on progression of the disease. This shows that FENG devices have started to be considered an effective medical diagnostic and treatment method by medical practitioners and that preclinical studies on participants have been conducted.

As the coronavirus disease 2019 (COVID-19) global pandemic has demonstrated, respiratory infections unquestionably have a profound impact on people’s lives. Wearing face masks that continuously track users’ breathing conditions could be beneficial for personalized healthcare and pandemic prevention because the characteristics of breathing and the symptoms of diseases vary from person to person.^141^ In 2022, Zhong et al.^129^ designed a wireless smart face mask by integrating an ultrathin self-powered pressure sensor and a compact readout circuit with a normal face mask (Figures 10C–10J). The developed pressure sensor, which served as the main component of this face mask, was a FENG device that was primarily made of two Au/parylene/Teflon AF films with four corners that are Au-Au bonded. With a total compressed thickness of 5.5 μm and a total weight of 4.5 mg, this FENG-based pressure sensor was fitted into conventional face masks and stimulated by low pressure. A peak open-circuit voltage of up to 10 V was obtained when airflow was stimulated, which allowed miniaturization of the readout circuit. Wireless monitoring and analysis of various breathing conditions, such as regular breathing, fast breathing, coughing, and breath holding, can also be achieved.

### Brain and bone injury

**Figure 11.**
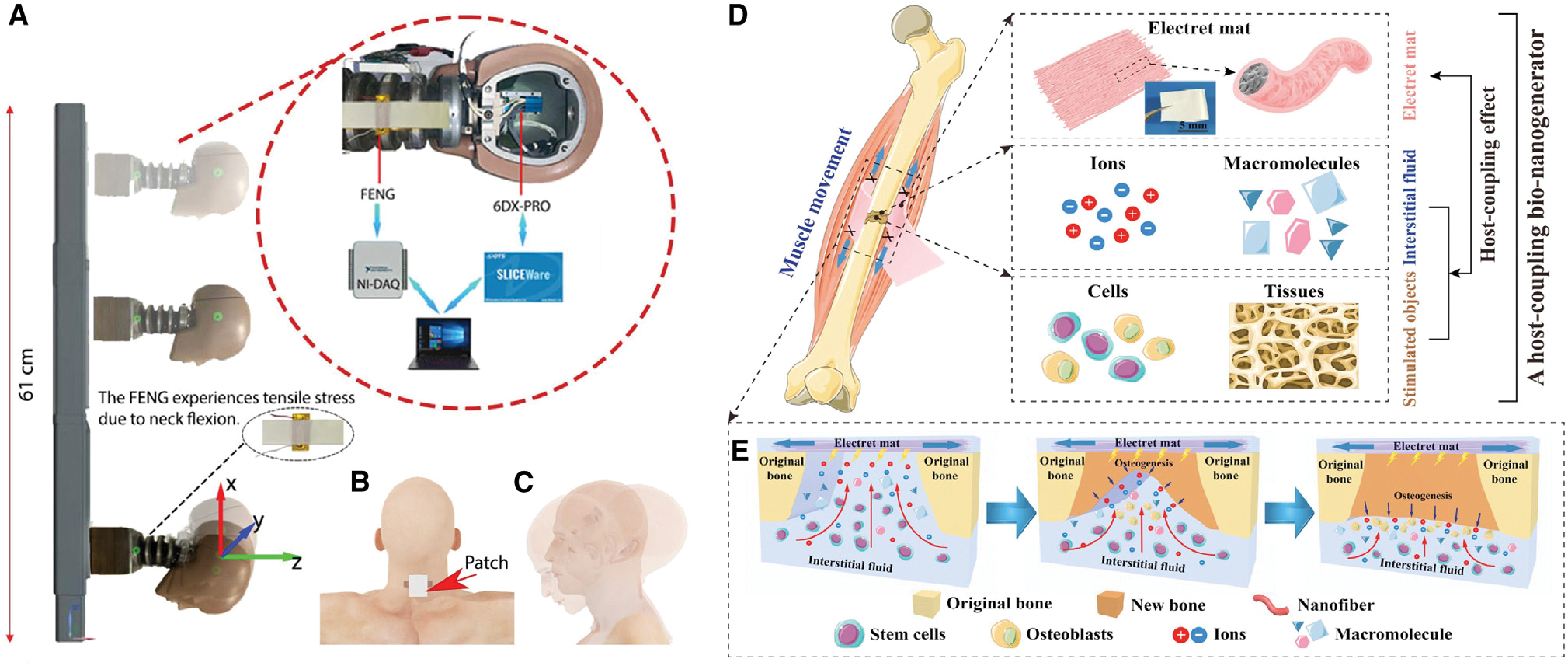
FENG-based personalized healthcare for brain injury prevention and bone injury recovery (A–C) Flexible, self-powered FENG sensor for estimating human head kinematics relevant to concussion. (A) A dummy was fixed to the center plate, then dropped in freefall. The inset shows the position of the 6-axis accelerometers inside the dummy head and the FENG sensor over the neck. (B) The location of the patch during implementation. (C) Head movement/kinematics “whiplash” that was characterized using the FENG sensor. Reproduced with permission.^136^ Copyright 2022, Springer Nature. (D and E) A host-coupling bio-FENG for electrically stimulated osteogenesis. (D) Schematic of the device implanted onto the bone injury *in vivo*. The device was made up of a FENG-based porous nanofiber mat and host to form a host-coupling effect. (E) Schematic showing the creation of electrical stimulation for triggering osteogenesis *in vivo* using scavenged biomechanical energy from muscle group movement. Reproduced with permission.^137^ Copyright 2021, Elsevier.

Concussion is a type of traumatic brain injury caused by a bump, blow, or jolt to the head or by a hit to the body that causes the head and brain to move rapidly.^142^ According to the Centers for Disease Control and Prevention (CDC) in the US, 20% of the estimated 1.7 million concussions each year are sports related.^143^ Aiming to estimate the human head kinematics relevant to brain injury in contact sports, in 2022, Dsouza et al.^136^ presented research on creation of a flexible, self-powered sensor patch based on a FENG to determine angular acceleration and angular velocity, the two essential indicators for concussion prediction (Figures 11A–11C). The developed device monitored the dynamic strain experienced by the neck through a thin PP-based FENG that produced a voltage pulse with a profile proportional to strain. Its capacity to transform mechanical energy into electrical output and its flexible, thin-film design make it a practical and advantageous choice for athletes in high-contact sports as a wearable patch. The results demonstrated that there was a strong positive correlation between the output of the FENG device and rotational kinematic signatures experienced by the human head, which were recorded using a triaxial accelerometer and gyroscope installed at the center of gravity of a human head dummy. This exemplifies the capability of obtaining an electronic signature that can be used to extract head kinematics during a collision and produce a marker to detect concussion.

To maintain physiological activity and function in the extracellular matrix (ECM), an endogenic electrophysiological microenvironment is essential.^144,145^ Particularly in cases of bone injury, cells’ behavior guidance is present under the influence of responsive bioelectric cues. Thus, mimicking bioelectricity could help treat bone injuries. Given that the need for batteries for *in vivo* power supply may increase the risk of infection and the likelihood of repeat surgery, in 2021, Yu et al.^137^ developed a host-coupling FENG to configure a self-powered regional electrical environment for powerful bone regeneration after injury (Figures 11D and 11E). The device was comprised of a FENG nanofiber mat coupled with interstitial fluid and stimulated objects of the host after implantation, forming a host-coupling effect. Biomechanical energy scavenging and electrical stimulation therapeutics were accomplished with this implantable FENG. According to the results, bone regeneration *in vivo* and osteogenesis differentiation of bone marrow mesenchymal stem cells were improved. Additionally, by controlling the electrical performance of the device, osteogenic ability was systematically assessed. When the developed FENG applied electrical stimulation, more cytosolic calcium ions were upregulated, which activated osteogenic differentiation and the calcium ion-induced osteogenic signaling pathway. This host-coupling FENG device offered explorative insight to aid development of electrical medical therapeutics.

### Pulse diagnosis and blood pressure

**Figure 12.**
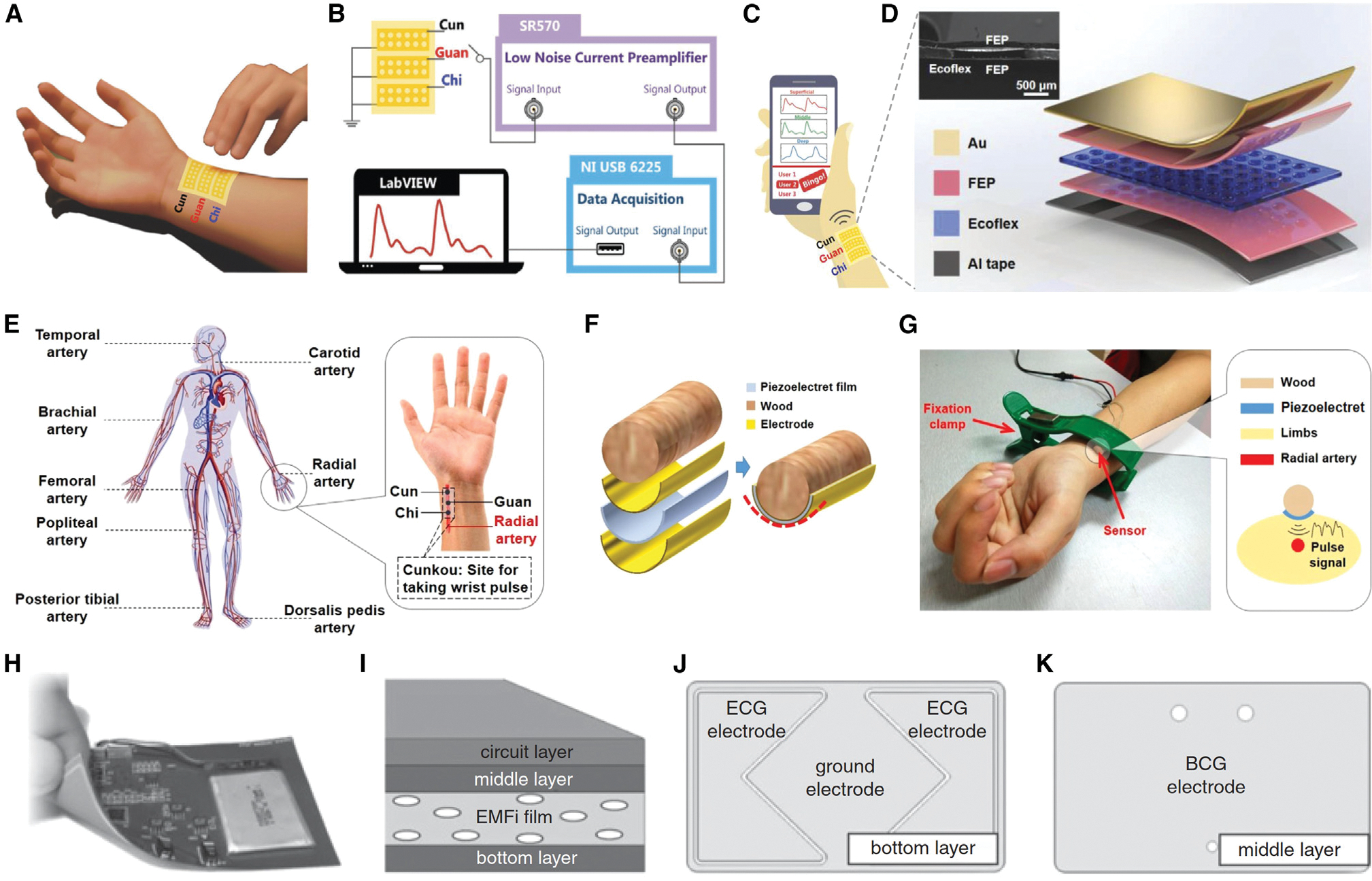
FENG-based personalized healthcare for pulse diagnosis and BP monitoring (A–D) Active pulse sensing system that can detect the weak vibration patterns of the human radial artery. (A) Three-finger pulse palpation at the Cun, Guan, and Chi positions. (B) Schematic of the diagnostic setup. (C) Concept of monitoring the pulse waves through a smartphone. (D) Schematic of the FENG-based pulse-sensing device. The inset shows a cross-sectional SEM image. Reproduced with permission.^138^ Copyright 2018, Wiley. (E–G) FENG-based pulse sensor combined with the ApEn analysis to detect human pulses. (E) Schematic of the distribution of the main arteries in the human body. (F) Structure of the pulse sensor with a cylinder-shaped wood piece to assist intimate contact between the sensor and the skin. (G) Pulse-sensing system with a mechanical fixture and a clamp used in the pulse-taking tests. Reproduced with permission.^139^ Copyright 2019, Elsevier. (H–K) A continuous BP monitor using the FENG-based patch-type sensor that simultaneously measured BCG and ECG. (H) Optical image of the FENG-based BP monitor. (I) Schematic of the cross-sectional view of the sensor. (J) Bottom layer electrodes for ECG measurement. (K) Middle layer electrodes for BCG measurement. Reproduced with permission.^135^ Copyright 2014, Wiley.

Pulse diagnosis is one of the essential tools used by many skilled medical professionals to identify potential problems with patient health. The appearance of the pulse, which is affected by dynamic pressure changes brought on by the heart’s contraction and relaxation and blood flow through the arteries, frequently reflects the health of the various organs and body parts as blood circulates throughout the body.^146^ Modern advancements in sensing technologies and the booming of AI have created a solid foundation for diagnostics of human pulses. Active pressure sensors based on FENGs offer an intuitive and sensitive way to detect pulse waves because they can faithfully and directly reflect the weak vibration of the radial artery for the pulse diagnosis. In 2018, Chu et al.^138^ created an active pulse-sensing system based on a FENG device with high equivalent piezoelectricity (*d*_*3*3_ ≈ 4,100 pC/N) that can recognize the faint vibration patterns of the human radial artery (Figures 12A–12D). Based on the FEP/Ecoflex/FEP sandwich structure, this active and flexible pulse-wave-sensing system had several important features, including excellent accuracy and stability, capability of identifying and categorizing pulses from various volunteers with the aid of Big Data analysis, detection of arrhythmia in volunteers who had previously received a diagnosis in hospitals using sophisticated and substantial ECG setups, viability of using a pulse-sensing system to measure and reveal blood pressure rather than a conventional blood pressure gauge, and imitation and recording of the standard three-finger pulse palpation signals. A later studies by Nie et al.^139^ investigated use of a FENG pulse sensor in conjunction with an approximate entropy (ApEn) analysis to detect the human pulse and reveal medical conditions (Figures 12E–12G). In this work, a flexible FENG (*d*_33_ ≈ 510 pC/N) with a wooden cylinder substrate was used to emulate the pulse-taking procedure needed to record the pulse characteristics. A group of 26 volunteers was diagnosed by the FENG-based wearable sensing system, and the ApEn analysis was implemented to analyze the data. Even though the proposed method cannot precisely identify the type of disease, it did offer a qualitative and helpful indicator to reflect the health condition of people. It is anticipated that future studies will improve the precision of human health condition diagnosis by utilizing more pulse data from more volunteers and AI technology.

As the “silent killer,” high blood pressure (BP) rarely has any symptoms most of the time. Frequent BP measurements can effectively prevent the progression of high BP, which otherwise will often remain unnoticed.^147,148^ Other than auscultatory and oscillometric methods that require a cuff and are inconvenient for continuous monitoring, BP can be estimated by the time it takes for a pulse pressure waveform to propagate between two points in the arterial tree.^149,150^ A continuous BP monitor was developed by Noh et al.^135^ (Figures 12H–12K) using a FENG-based patch-type sensor that simultaneously measured the BCG and ECG. The device was fabricated as a layered structure based on the FENG thin film, along with screen-printed electrodes and a flexible electronic circuit. The ground electrode served as a reference point for the ECG measurement, which involved recording the potential difference between the two ECG electrodes situated in the bottom conductive layer. When measuring the BCG, the electric current generated by the FENG was measured at the BCG electrode, which was positioned in the middle conductive layer. In addition, a flexible electronic circuit was integrated as a top layer for conditioning the ECG and BCG waveforms. Three adult subjects underwent testing, and the results showed that the estimated and reference SBPs had excellent agreement. Moreover, the performance met the criteria of the US Association for Advancement of Medical Instrumentation (AAMI) and the British Hypertension Society (BHS).

### Medical imaging

**Figure 13.**
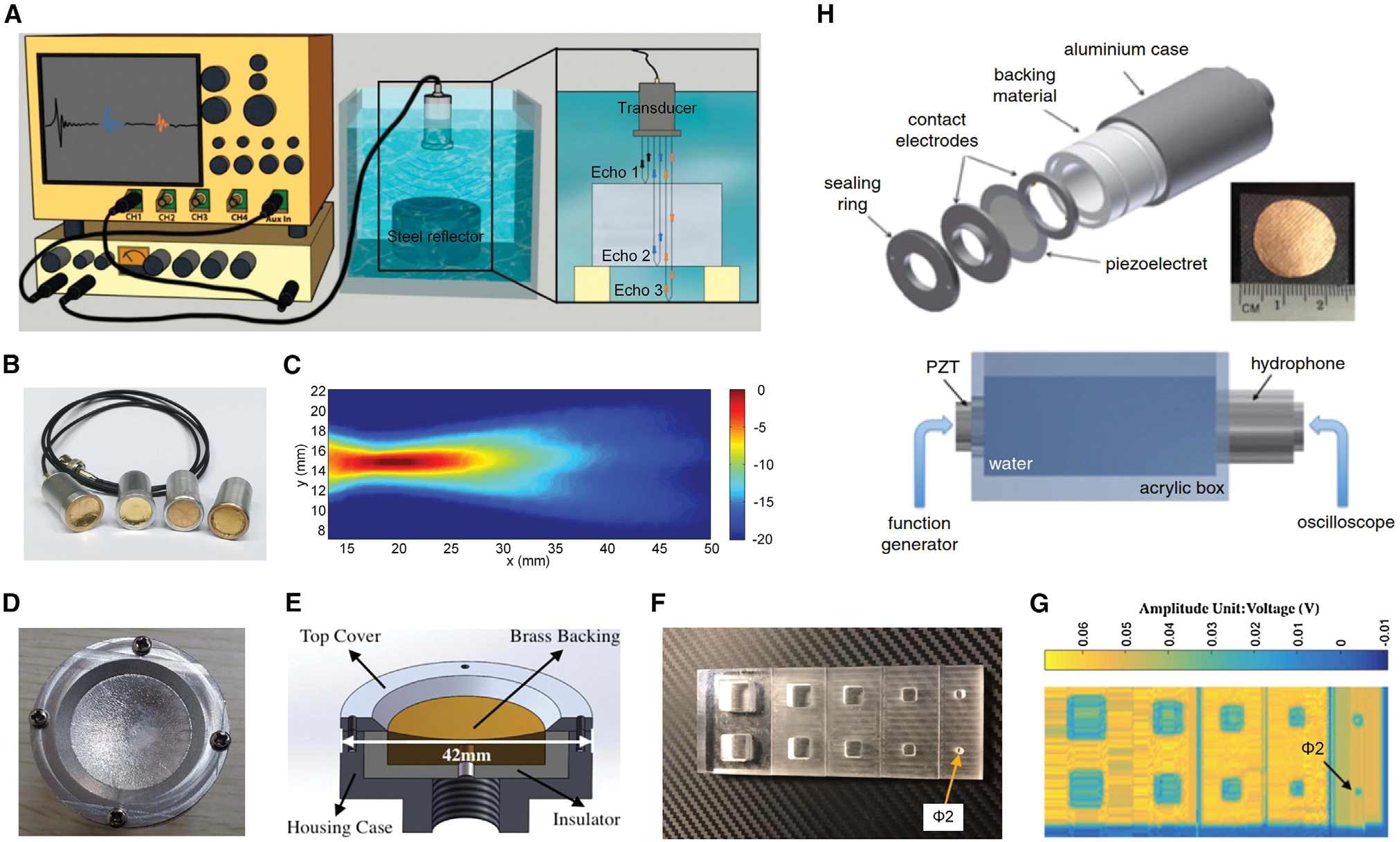
FENG-based personalized healthcare for medical imaging (A–C) FENG-based ultrasonic transducers for pulse-echo water immersion for quantitative echography. (A) Schematic of the experimental setup. (B) Photographs of the fabricated transducer prototypes. (C) The measured axial acoustic field distribution for a fabricated transducer. Reproduced with permission.^27^ Copyright 2020, MDPI. (D–G) ACU transducer made of PP FENG for non-contact ultrasonic imaging. (D and E) Optical image (D) and schematic (E) of the cross-section of the spherically focused transducer. (F) A scanned sample with five steps and square sink holes. (G) Ultrasonic imaging of the square-sink-hole sample. Reproduced with permission.^155^ Copyright 2019, Elsevier. (H) Exploded view of the hydrophone with the 3D-printed PP FENG (top part) and schematic of the measurement setup with a PZT acoustic emitter and signal detector (bottom part). Reproduced with permission.^140^ Copyright 2019, Wiley.

Medical imaging is used to view the human body to diagnose, monitor, or treat medical conditions.^151,152^ Among different technologies, ultrasound imaging is a widely used medical imaging tool that has an excellent safety record.^153^ For ultrasound imaging and quantitative echography, where spectral analysis of reflected pulses can reveal the structure and movement of the body’s internal organs as well as blood flowing through the vessels, ultrasonic transducers with a short impulse response and a wide frequency band response are desirable. The electromechanical-acoustical properties of FENGs recently opened up new avenues for the design and development of ultrasonic transducers.^40,154–157^ To shed some light on how to enhance FENGs’ properties for water-coupled ultrasonic inspection, Quirce et al.^27^ presented the pulse-echo response of immersed FENG-based ultrasonic transducers (Figures 13A–13C). Prototypes that were flat and spherically focused were constructed and tested, and different thin polymeric coatings intended to shield the transducers’ surface were tried. The results demonstrated that, despite the reduced sensitivity, the extremely wide band response (−6 dB band from 0.29–2.7 MHz) still makes this type of ultrasonic transducer useful for various water immersion applications, including materials characterization, nondestructive tests, and medical imaging, where large bandwidth, moderate frequencies (0.5–4.0 MHz), and good axial resolution are required. Moreover, FENGs’ characteristics, such as low acoustic impedance, strong piezoelectric response, high flexibility, low density, and wide bandwidth, build on their potential for air-coupled ultrasound (ACU) applications. Currently, most hospitals still use coupling media, like water or gel, during ultrasonography to lower the acoustic impedance. Tang et al.^155^ developed planar and spherical ACU transducers using a PP FENG with a thickness of 55–60 μm and a piezoelectric coefficient *d*_33_ of 200 pC/N as the transducing element (Figures 13D–13G). The spherical ACU transducer had an aperture of 30 mm, a radius of curvature of 40 mm, and a focusing range of 38–43 mm. In the ultrasonic imaging experiment, an acrylic square-sink-hole sample with five steps and two sinkholes on each step was scanned. The transducer was mounted on a platform that was moved along the x and y axes by two servomotors. Scanned images showed that the shape was well displayed and the steps separating the terraces could be distinguished. Additionally, it was demonstrated recently that 3D printing is a good substitute fabrication method for manufacturing FENG-based ultrasonic transducers for medical imaging.^120,140,158^ For example, Palito et al.^140^ presented a transducer for ultrasound imaging based on a 3D-printed PP FENG (Figure 13H). The validation was performed using a PZT piezoelectric ceramic submerged in water to generate ultrasonic sweeps that were sensed by the 3D-printed ultrasonic transducer. The tests demonstrated that the transducer’s sensitivity can reach up to 600 mV, and it can precisely detect the PZT’s acoustic resonance frequency at 43.7 kHz.

## ANIMAL AND PLANT APPLICATIONS

Aside from studies on human-related research that include advancing human-machine interfaces, harvesting mechanical energy from the human body, and promoting the development of personalized healthcare as mentioned above, FENGs have also demonstrated their potential in animal and plant-related applications, extending their bioengineering applications beyond human beings. This section introduces some progress in FENG applications for development of an artificial bat head that can detect obstacles,^42^ assessment of spawning sea lamprey migration,^159,160^ and diagnostic systems that can detect the internal structure of wood.^161–163^

**Figure 14.**
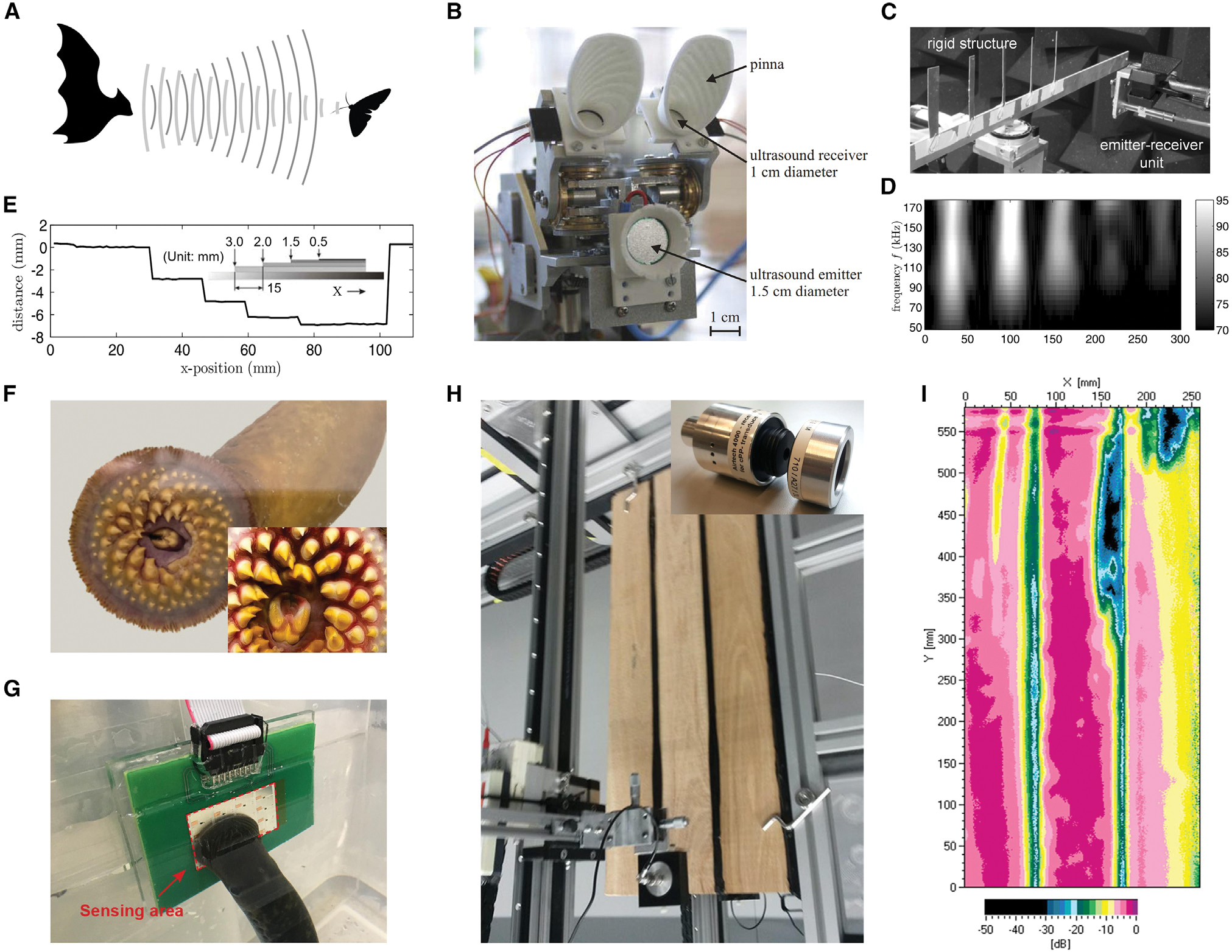
FENG for animal and plant applications (A–E) FENG for development of an artificial bat head. (A) Schematic showing the bat using echolocation to navigate and hunt. (B) Photograph of the artificial bat head. (C) Measurement setup for determination of the lateral spatial resolution. (D) Waterfall diagrams of the reflected SPL. (E) Measured height profile of the stair structure. Reproduced with permission.^42^ Copyright 2011, IEEE. (F and G) FENG for underwater invasive animal detection. (F) Photographs of an adult sea lamprey. The inset shows a magnified photo of the mouth of the sea lamprey. (D) Photograph of a sea lamprey attaching to the FENG-based sensing panel in an underwater environment. Reproduced with permission.^160^ Copyright 2022, Springer. (H and I) ACU detection of natural defects in red oak samples using a FENG in transmission mode. (H) Photograph of the scanning measurement system. The inset shows a photograph of the FENG receiver. (I) C-scan attenuation image measured at a frequency of 100 kHz. Reproduced with permission.^162^ Copyright 2020, Springer.

To enable functional replication of the biosonar system found in bats (Figure 14A), Rupitsch et al.^42^ developed an artificial bat head based on ultrasonic transducers that were made of FENGs. The cellular structure of FENGs allows them to have a high piezoelectric strain constant (*d*_33_ ≈ 600 pC/N) and a wide bandwidth (20–200 kHz). The artificial bat head (Figure 14B) comprised two ultrasound receivers (diameter of 1.0 cm) and an ultrasound emitter (diameter of 1.5 cm) that were all made using FENGs. The reflected sound waves were concentrated via rotatable pinnae. The overall acoustic system of the artificial bat head was characterized; it consisted of a signal generator, high-voltage amplifier, emitter, receiver, and signal amplifier. Lateral spatial resolution measurement was conducted using a translation unit, and the forks were traversed with respect to the emitter-receiver unit (Figure 14C). When the emitter was excited by a sine-burst signal (10 periods; U_pp_ = 600 V) with different frequencies, the measurement result was obtained for varying × positions at a distance of 20 cm between the emitter-receiver unit (Figure 14D). This waterfall diagram shows that a higher burst frequency was accompanied by a better geometric resolution. Additionally, a stair structure was used to determine the axial spatial resolution, which was decisive for distance measurements (Figure 14E). By measuring the time of flight of the propagating acoustic waves, the distance between the artificial bat head and the obstructions was calculated. When a sine-sweep excitation with frequencies ranging from 20–300 kHz served as the emitter’s input signal, the artificial bat head was able to achieve an axial spatial resolution of 0.1 mm.

FENG technology can also be applied for detection of sea lampreys, an invasive underwater species that has greatly harmed native fish communities, leading to establishment of a binational, basin-wide population control program.^164^ In 2022, Cao et al.^160^ demonstrated a species-specific nondestructive sensing system based on FENGs for *in situ* monitoring of the sea lamprey’s spawning phase during migration. The sea lamprey is one of the very few fish species that possess oral suction (Figure 14F), which can be used to find this aquatic invader. Because of the foamed structure, FENGs have demonstrated a certain ability of elastic deformation that enables them to respond to positive and negative pressure.^159^ The developed pressure sensor array based on FENGs converted mechanical stimuli into electrical signals under positive and negative pressure. The performance was experimentally validated by quantitative analysis in a wide pressure range of −50 to 60 kPa. For the sea lamprey detection, a 4 × 4-pixel sensor array was developed and integrated with a complementary metal-oxide-semiconductor (CMOS)-based signal processing array (Figure 14G). This sensing strategy employed a series of pumping actions as the species-specific biological footprint rather than a single negative pressure pulse, aiming to avoid possible false reading signals from other marine animals and improve the sensing reliability for field deployment. In this way, FENGs can enable a robust and cost-efficient solution for *in situ* nondestructive evaluation of invasive sea lampreys.

Through-transmission scanning measurements can be conducted through FENG-based ultrasonic transducers to detect natural features of wood, such as knots, cracks, grain orientation, or annual rings.^41,161–163^ Other methods, like radiography, can only be performed with extremely high safety precautions, whereas those using electrical resistivity or ultrasound require contact or a couplant between the transducer and the wood. It has been demonstrated that FENG-based ultrasonic transducers with a high signal-to-noise ratio can enable internal structure recognition of conventional timber, glued laminated timber, plywood, etc. in transmission mode.^161^ The FENG-based ultrasonic transducer was made of cellular PP and had a high signal-to-noise ratio for ACU testing.^161–163,165^ The transmission of ultrasonic waves between the transducer and air is made possible by a small difference in acoustic impedance caused by the FENG’s low elastic modulus and low density. Because of the non-contact measurement, large areas can be automatically scanned, and defects can be quickly and graphically identified. For example, Tiitta et al.^162^ conducted research where a nondestructive method based on FENGs was used for wood analysis. This study employed a FENG ultrasonic transmitter that was excited with 1.8-kV unipolar pulses so that the electrostatic compression of the FENG made a significant contribution to its vibration. Measurements were conducted for oak and spruce samples to determine the relationships between the responses and the wood defects. After the measurement series, the samples were cut from the measurement regions, and the results of the visual examination of the cross-sections were in agreement with the ultrasound responses. Furthermore, different from the transmission mode in that the sample was placed between the transmitter and receiver, FENG transducers can be used in reflection mode, where the transmitter and receiver are on one side of the specimen.^163^ Although measurement in reflection is challenging because the signal was weak as a result of scattering and interfering waves on the sound path, millings, cavities, and drillings can still be detected and localized. Time of flight can also be used to gauge the depth of the flaws. When only one side of the tested object is accessible or the depth ranges of the features are of interest, this kind of measurement is preferred at the expense of reduced measuring accuracy and penetration depth.

## SUMMARY AND PERSPECTIVE

As an intriguing invention that first began to take off around the turn of the millennium, FENGs have emerged from their infancy step by step and started to reveal their application prospects, particularly in various fields of bioengineering. An unprecedented favorable environment for practical application and industrialization of FENG technology has been created by the rapid advancement of the IoE, Big Data, and 5G/6G. With the abundance of research efforts on FENGs for bioengineering, FENGs have been ushering in the fast development of bioengineering applications in breadth and depth. Scrutinizing the literature reveals that FENG research has grown by leaps and bounds in the last decade. Academia has seen the development of many different FENG materials, devices, and fabrication techniques; Table 1 lists some recent examples.

From booming wearable sensors to thriving intelligent robots, the exchange of information between humans and machines through HMI will have a profound impact on human society. Because of their intrinsic properties that allow bidirectional reciprocal flow of information and energy, FENGs may make a special contribution to the upcoming IoE era, underscoring the interaction between humans and machines. We classify this trend into four groups: tactile perception, voice interaction, body movement monitoring, and footstep tracking. Another point is that FENGs might alter the future of wearable/implantable devices by supplementing them with bioenergy-derived power, possibly making them self-powered. Providing them with renewable energy sources, this feature may aid restructuring of current electronics. We categorize the current advancements in FENG-based biomechanical energy harvesting into different forms: pressure bioenergy harvesting (aiming at pressurized form), vibration bioenergy harvesting (aiming at vibration form), textile-based bioenergy harvesting (aiming at *in vitro* haphazard form), and implantable ultrasonic energy harvesting (aiming at *in vivo* energy transmission). Moreover, personalized healthcare is advancing with the current technology revolution to change people’s lives, offering an opportunity for new technologies to get involved. FENGs are actively participating in this race to provide good healthcare solutions and are moving in the direction of the next generation of personalized healthcare. With the help of FENGs, health and fitness can be readily monitored and tracked for regular people using wearables or smart clothing; patients can be diagnosed remotely, leading to a significant reduction in hospital visits; athletes can get better sports monitoring and protection, preventing permanent post-exercise injuries; and those at risk of chronic diseases like hypertension or AD can readily get examination and early intervention. Apart from these, FENGs are also bringing new technological impetus to medical imaging, which is critical for saving lives and enhancing life quality. Last but not least, by introducing FENG applications related to animals and plants, we want to highlight the fact that FENG research not only contributes to bionic engineering that will benefit and advance human technology but also enables us to understand and protect other species in a more scientific way, which, in turn, will benefit our entire ecosystem.

Although the bioengineering application of FENGs has gradually advanced past the feasibility analysis stage and is now heading toward a rapid development phase driven by a variety of practical applications, several possible challenges still deserve the attention of researchers to advance extensive applications and enhance the value of FENGs in bioengineering applications. Here are some of the current bottlenecks of this technology with a brief summary.

### High voltage and low current

FENGs share one characteristic with piezoelectric materials: high impedance, which results in high output voltage and low output current. It is typical for FENGs to obtain open-circuit voltage spikes around 100 V but short-circuit currents of several microamperes. The issue could be alleviated by proposing an increase in the surface area of FENG thin films or by stacking single layers to form multiple layers, which does not represent a major burden because a single-layer FENG readily attains a thickness of less than 100 μm. In addition, appropriate postprocessing treatment of FENGs (e.g., thermal pressure treatment^170,185^) is a useful way to enhance their performance. A different tactic is to modify the design and fabrication of FENGs from scratch. This involves investigating the utilization of different polymers with special characteristics for building cellular structures; adopting novel fabrication techniques to optimize the size, shape, arrangement, or density of the artificial voids; and managing to raise the inner surface charge density of the voids. Furthermore, FENG performance is expected to take a step forward with a novel design of high-efficiency power management circuitry that would increase current at the expense of output voltage; for example, by utilizing different voltage step-down power electronic circuit configurations.

### Dynamic wearability

In the design of FENG devices, dynamic wearability (including the device’s ability to deform and conform while the body is in motion) is not always a top priority, but its lack can hinder long-term usage. Although FENGs’ thin-film structure and internal foam make them lightweight, the metal coating may negatively impact performance, durability, and softness. To address this, a solution could be to use lighter, more flexible materials like CNT fiber electrodes^186^ or fabric-based electrodes.^47,48^ Additionally, FENG devices with curved surfaces are harder to fabricate and can cause wrinkles and performance degradation when stretched. A fully stretchable FENG with stable performance is desired to improve dynamic wearability and broaden its uses in e-skin and e-tattoo applications. An additional advantage of good stretchability is the improved transverse sensitivity of FENGs, making them suitable for applications requiring large deflections. Breathability is also important for dynamic wearability and to prevent skin issues like rash and excessive sweating. Solutions may include use of natural or biocompatible materials or adding breath holes through microfabrication.

### Performance decay

FENG devices show some degree of deterioration in their electromechanical response performance over time.^187^ It is not unusual for performance to drop by about 50% from the initial performance in the first 30 days. This drawback is primarily brought on by the loss of positive and negative charges on the internal surfaces of the artificial voids inside the FENG. Studies conducted over the course of the life cycles of FENG devices could be valuable because they may need to operate for years or even longer if their self-powered capability is anticipated to be fully utilized. Use of additional processing procedures, such as charging and pre-aging at high temperatures,^188,189^ could help enhance long-term performance as well. Additionally, more investigation into new materials and micro/nanostructures on the inner surface of the void may provide new insight into stabilizing FENG performance over their lifetime. It is also essential to consider the impact of environmental factors, such as temperature and humidity, on FENG performance. Proper encapsulation is another strategy to help preserve residual charges and ensure stability. The performance decay caused by mechanical degradation should not be overlooked because abrasion and mechanical failure may gradually worsen FENG performance and affect their long-term application.

### Biodegradability

Biodegradability is worth consideration in the development of FENG devices.^190^ Use of biodegradable sensors in certain medical applications, such as wound or bone healing, can eliminate the need for invasive implant removal and reduce the risk of infection or tissue damage. Moreover, mass production of FENG devices to meet the demands of the IoE will result in significant waste if not properly managed. By fabricating FENG devices entirely or partially out of biodegradable materials, this issue can be effectively addressed. A range of biocompatible, biodegradable, and bioabsorbable substances, including natural products such as silk, wool, and cotton and synthetic compounds like peptides, polylactide, polyamide, and polyester, have been identified as potential solutions. For instance, a recent FENG device^187^ was made with polylactide, which is biodegradable throughout its life cycle, balancing eco-friendliness and performance. It is likely that further advancements in the creation of biodegradable FENG devices will emerge in the near future.

### Self-healing

FENG devices inevitably deteriorate over time because of use-related wear, warping, or stretching as well as damage from physical fatigue or chemical agents. Self-healing materials have become available for building robust and self-healing electronic devices,^191^ enabling wearable devices to autonomously heal from cracks and restore their mechanical and electrical properties. As of today, a variety of self-healing materials, their compositions, healing mechanisms, and potential applications in wearable devices have been explored.^192^ These efforts lay a strong foundation for the creation of self-healing FENG devices, allowing them to withstand wear and tear, reduce maintenance costs, and act, to some extent, like a living organism. Incorporating self-healing capabilities into the composition of FENG devices will enhance their durability and longevity, making them a smart choice for long-term applications, particularly in the field of bioengineering.

## Figures and Tables

**Table 1.** Piezoelectric *d*_33_ coefficient of various FENGs with their producing methods

Production methods	Materials	Reported *d*_33_ (pC/N)	Thickness (μm)	Charging conditions	Year	Reference

Foaming	PP	200	150	−16 kV for 3 min	2015	Wu et al.^166^
Foaming	PP	175	100	–	2014	Anton et al.^104^
Foaming	PP	400	80	–	2020	Wan et al.^67^
Foaming	IXPP	650	183	−25 kV for 60 s	2015	Zhang et al.^167^
Foaming	PP/CaCO_3_	800	–	−21 kV for 60 s	2016	Mohebbi and Rodrigue^168^
Foaming	PVDF/CaCO_3_/MTM	251	80–100	27 kV for 10 min	2019	Jahan et al.^169^
Foaming	PE	2,550	600	−21 kV	2018	Hamdietal.^170^
Hot-pressing	FEP/f-PTFE	6,380	250	−15 kV for 3 min	2017	Wang et al.^171^
Hot-pressing	EVA/BOPP	–	125	−18 kV for 5 min	2016	Zhong et al.^107^
Hot-pressing	FEP	3,700	500	5 kV	2014	Zhang et al.^172^
Hot-pressing	PET/EVA	6,300	1,000	−20 kV	2017	Zhong et al.^173^
Evaporation	Yb^3+^/PVDF	−362.9	170	self-polarization	2016	Ghosh et al.^52^
Evaporation	Pt NP/PVDF	−686	150	self-polarization	2015	Ghosh et al.^53^
Evaporation	PVDF/Nafion	–	500	self-polarization	2017	Xu et al.^174^
Evaporation	Mg salt/PVDF	–	300	self-polarization	2015	Adhikary et al.^175^
Evaporation	PVDF/3D MOF	143	–	self-polarization	2020	Roy et al.^176^
Microfabrication	parylene-C	30,000	–	soft X-ray charging	2019	Lu and Suzuki^177^
Microfabrication	PDMS	520	200	−25 kV for 2 min	2019	Shietal.^178^
Microfabrication	PDMS	350	150	4 kV at 0.5 Hz	2016	Kachroudi et al.^179^
Mechanical assembly	FEPtube	160	600	±6 kV	2018	Zhukov et al.^118^
Mechanical assembly	FEP/polymer foam	1,000	1,000	−30 kV 60 s	2018	Shi et al.^180^
Mechanical assembly	PTFE/porous PTFE	1300	100	–	2019	Chen et al.^181^
Perforation	FEP/Ecoflex	4,050	150	−18 kV	2019	Zhong et al.^25^
Perforation	FEP/PVB	4,680	75	±20 kV for 5 min	2022	Wan et al.^128^
Thermal phase separation	PP/PZT	–	80	−18 kV for 60 s	2018	Yan et al.^91^
Laser cutting	FEP/Ecoflex	4,100	210	−18 kV	2018	Chu et al.^138^
Freeze casting	PVDF	264	1,500	26.8 kV for 30 min	2019	Zhang et al.^182^
3D printing	ABS	87	1,000	8 kV	2020	Kierzewski et al.^120^
Cellular free	FEP/PVA	930	25	16 kV	2021	Xu et al.^183^
Bubble generation	PDMS	206	3,000	25 kV for 2 min	2021	Zhang et al.^184^

IXPP, irradiation cross-linked polypropylene; MMT, montmorillonite; CaCO_3_, calcium carbonate; PE, polyethylene; Yb^3+^, ytterbium; NP, nanoparticle; MOF, metal-organic framework; PVB, polyvinyl butyral; ABS, acrylonitrile butadiene styrene.
